# Human Cytomegalovirus RNA2.7 Is Required for Upregulating Multiple Cellular Genes To Promote Cell Motility and Viral Spread Late in Lytic Infection

**DOI:** 10.1128/JVI.00698-21

**Published:** 2021-09-27

**Authors:** Betty Lau, Karen Kerr, Salvatore Camiolo, Katie Nightingale, Quan Gu, Robin Antrobus, Nicolás M. Suárez, Colin Loney, Richard J. Stanton, Michael P. Weekes, Andrew J. Davison

**Affiliations:** a MRC-University of Glasgow Centre for Virus Research, Glasgow, United Kingdom; b Cambridge Institute for Medical Research, University of Cambridge, Cambridge, United Kingdom; c Division of Infection and Immunity, Cardiff University School of Medicine, Cardiff, United Kingdom; Northwestern University

**Keywords:** cell motility, human cytomegalovirus, lncRNA, regulation of gene expression

## Abstract

Long noncoding RNAs (lncRNAs) are frequently associated with broad modulation of gene expression and thus provide the cell with the ability to synchronize entire metabolic processes. We used transcriptomic approaches to investigate whether the most abundant human cytomegalovirus-encoded lncRNA, RNA2.7, has this characteristic. By comparing cells infected with wild-type virus (WT) to cells infected with RNA2.7 deletion mutants, RNA2.7 was implicated in regulating a large number of cellular genes late in lytic infection. Pathway analysis indicated that >100 of these genes are associated with promoting cell movement, and the 10 most highly regulated of these were validated in further experiments. Morphological analysis and live cell tracking of WT- and RNA2.7 mutant-infected cells indicated that RNA2.7 is involved in promoting the movement and detachment of infected cells late in infection, and plaque assays using sparse cell monolayers indicated that RNA2.7 is also involved in promoting cell-to-cell spread of virus. Consistent with the observation that upregulated mRNAs are relatively A+U-rich, which is a trait associated with transcript instability, and that they are also enriched in motifs associated with mRNA instability, transcriptional inhibition experiments on WT- and RNA2.7 mutant-infected cells showed that four upregulated transcripts lived longer in the presence of RNA2.7. These findings demonstrate that RNA2.7 is required for promoting cell movement and viral spread late in infection and suggest that this may be due to general stabilization of A+U-rich transcripts.

**IMPORTANCE** In addition to messenger RNAs (mRNAs), the human genome encodes a large number of long noncoding RNAs (lncRNAs). Many lncRNAs that have been studied in detail are associated with broad modulation of gene expression and have important biological roles. Human cytomegalovirus, which is a large, clinically important DNA virus, specifies four lncRNAs, one of which (RNA2.7) is expressed at remarkably high levels during lytic infection. Our studies show that RNA2.7 is required for upregulating a large number of human genes, about 100 of which are associated with cell movement, and for promoting the movement of infected cells and the spread of virus from one cell to another. Further bioinformatic and experimental analyses indicated that RNA2.7 may exert these effects by stabilizing mRNAs that are relatively rich in A and U nucleotides. These findings increase our knowledge of how human cytomegalovirus regulates the infected cell to promote its own success.

## INTRODUCTION

Long noncoding RNAs (lncRNAs) are untranslated transcripts of over 200 nucleotides (nt). More than 16,000 have been annotated in the human genome (Human GENCODE Release, version 30), and many that have been studied in detail have been shown to be vital regulators of cellular processes. The functional importance of lncRNAs is also evident from the association of their dysregulation with over 300 human diseases (LncRNADisease database [1]) and from loss-of-function experiments targeting lncRNAs in animal models that result in embryonic lethality or developmental defects (2). Cellular lncRNAs function in various ways, including guiding lncRNA-bound proteins to specific DNA sequences, acting as decoys for proteins or miRNAs, forming scaffolds for multiprotein complexes that would otherwise be thermodynamically unfavorable, altering RNA processing or translation directly by binding to DNA or RNA or indirectly by modulating the activity of regulatory proteins, and modulating signaling pathways (2–5).

Herpesviruses also encode lncRNAs that regulate multiple processes during infection. Kaposi’s sarcoma-associated herpesvirus polyadenylated nuclear (PAN) RNA is essential for late gene expression and viral propagation (6–8). Herpes simplex virus 1 latency-associated transcript (LAT) influences various stages of latency (9). Human cytomegalovirus (HCMV; species *Human betaherpesvirus 5*) encodes four major lncRNAs (RNA1.2, RNA4.9, RNA5.0, and RNA2.7), in addition to at least 170 coding sequences (CDSs) for functional proteins (10–13). RNA1.2 suppresses extracellular release of the proinflammatory cytokine interleukin-6 (IL-6) by blocking NF-κB activation (14). RNA4.9 has been reported to recruit the polycomb repression complex to suppress the activity of the HCMV major immediate early promoter (MIEP) via chromatin remodeling (15), which could contribute to the establishment and maintenance of latent infection. RNA4.9 also facilitates lytic infection, forming an RNA-DNA hybrid (R-loop) with the origin of DNA replication (*oriLyt*) that may be involved in the initiation of viral DNA synthesis (16).

RNA2.7 is approximately 2.5 kb in size (not including the polyadenylate tail) and is encoded by a gene that does not overlap known functional CDSs. It is relatively A+U-rich (55%) and well conserved (78% identity in all sequenced strains), although both of these values are somewhat lower in the 300 to 400 nt at the 5′ end (data not shown). The gene is transcribed with early kinetics and thus dependent on prior immediate early gene expression. It is activated by immediate early proteins 1 and 2 (IE1 and IE2, encoded by genes UL123 and UL122, respectively), and other viral gene products are also required for the extremely high levels of RNA2.7 expression during lytic infection (17). RNA2.7 has long been known to be the most abundant early transcript (18–21) and constitutes nearly half of all viral polyadenylated transcripts late in infection (22). Moreover, it is highly expressed in multiple cell types (fibroblasts, dendritic cells, and macrophages) during lytic infection (22, 23), and there is evidence that it is also abundant during latency (24).

RNA2.7 interacts with the GRIM-19 subunit of mitochondrial complex I (also known as respiratory complex I, NADH:ubiquinone oxidoreductase, and type 1 NADH dehydrogenase), which is the first component in the mitochondrial electron transport chain, leading to the maintenance of ATP production and the protection of infected cells from apoptosis (25). Capitalizing on this activity, RNA2.7 protected endothelial cells from apoptosis in an *in vitro* model of ischemia/reperfusion injury (26) and has been administered successfully as a therapeutic treatment to prevent and rescue neuronal death in animal models of Parkinson’s disease (27). Given that the 796-nt sequence forming the 5′-terminal one-third of RNA2.7 (termed the p137 region) was sufficient to protect cells from apoptosis (27), it is likely that the rest of this lncRNA performs additional roles. Observations consistent with this hypothesis have been reported, in that RNA2.7 is distributed throughout the nucleus and the cytoplasm, whereas mitochondrial complex I is located only in the mitochondrial membrane (16, 28), and in that protection of cells from apoptosis is unlikely in principle to require such high levels of RNA2.7. In the present study, we investigated the activities of RNA2.7 further by characterizing the properties of two RNA2.7 deletion mutants, with an emphasis on transcriptome profiling. RNA2.7 was required for regulating hundreds of cellular genes, and >100 of these genes were associated with promoting the movement of infected cells late in infection. The observation that upregulated transcripts tend to be A+U-rich and contain sequence motifs associated with RNA instability suggests that RNA2.7 may be involved in promoting mRNA stability.

## RESULTS

### Analysis of growth kinetics.

**FIG 1:**
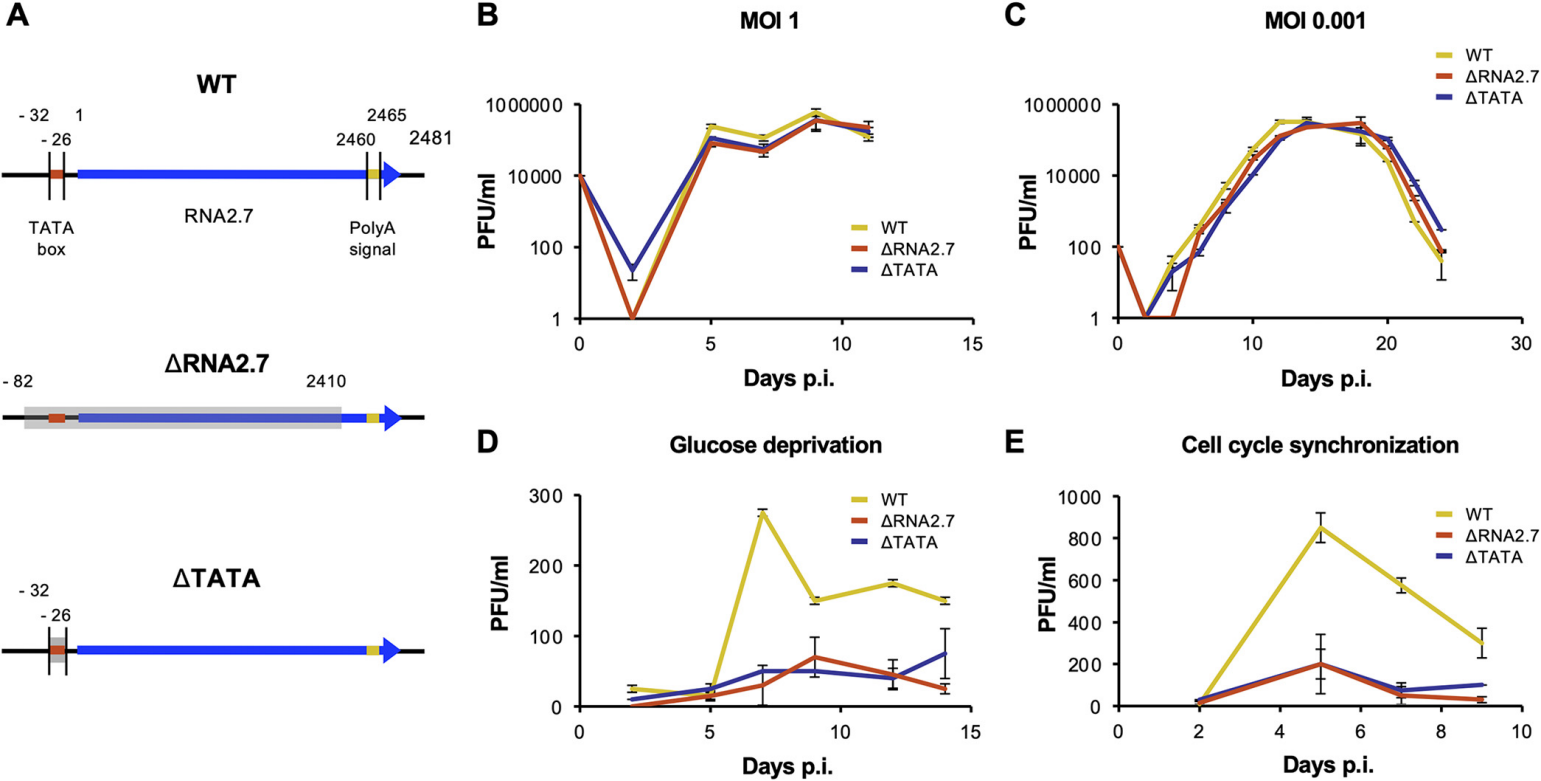
Effects of RNA2.7 on viral growth kinetics. (A) Diagram depicting the regions deleted in the RNA2.7 mutants (ΔRNA2.7 and ΔTATA) in comparison with WT. The RNA2.7-coding region is indicated by a blue arrow, the TATA box by a red line, the polyadenylation [poly(A)] signal by a yellow line and deleted regions by gray-shaded rectangles. Numbers indicate the distance (nt) from the transcription initiation site (at 1 nt). In the HCMV genome, the RNA2.7 gene is oriented leftward near the left end (22). For analysis under standard culture conditions, HFFF2 cells were infected at an MOI of  1 (B) or an MOI of  0.001 (C) with WT, ΔRNA2.7, or ΔTATA, and cell-released viral titers were determined. For analysis under conditions of glucose deprivation (D) or cell synchronization (E), HFFF2 cells were infected with WT, ΔRNA2.7, or ΔTATA at an MOI of  5. Each result represents one of three independent experiments, with each error bar indicating the standard deviation of technical replicates. PFU, plaque-forming units.

The growth kinetics of three viruses generated from a bacterial artificial chromosome (BAC) of HCMV strain Merlin were examined: the parental virus (termed wild type [WT]) and two RNA2.7 deletion mutants (ΔRNA2.7 and ΔTATA) (Fig. 1A). In ΔRNA2.7, the majority of the RNA2.7 coding sequence was deleted, from 50 bp upstream of the TATA box (TATAAA) to 50 bp upstream of the polyadenylation signal (AATAAA) that RNA2.7 shares with an upstream gene, RL5A. In ΔTATA, only the 6 nt of the TATA box were deleted.

A phenotype has been reported for an RNA2.7 deletion mutant of HCMV strain Toledo (29), and the genome sequence of this mutant has been verified (13). The growth kinetics of this mutant were wild type under standard culture conditions but inhibited under conditions of glucose deprivation. This phenotype was attributed to the increased susceptibility of mutant-infected cells to apoptosis and their decreased production of ATP (25). The ability of the Merlin RNA2.7 mutants to grow in human fetal foreskin fibroblast (HFFF2) cells was examined under conditions of single-step and multistep infection (Fig. 1B and C). Neither mutant showed a growth defect in cells under standard culture conditions, but both grew poorly under conditions of glucose deprivation, thus confirming with strain Merlin the earlier findings with strain Toledo (Fig. 1D). Both mutants also exhibited a growth defect in HFFF2 cells synchronized to the G_0_ phase of the cell cycle (Fig. 1E).

### Transcriptomic analysis.

Since many functions attributed to lncRNAs involve regulation of gene expression, differential expression of viral and cellular transcripts was examined in HFFF2 cells lytically infected with the RNA2.7 mutants in comparison to cells infected with WT. Illumina sequence data were obtained at three time points postinfection (p.i.): 4 h p.i. for the effects of RNA2.7 incorporated into input virions (15, 30) or expressed very early in infection, and 24 and 72 h p.i. for the effects of RNA2.7 expressed during the early and late stages of infection, respectively.

**FIG 2:**
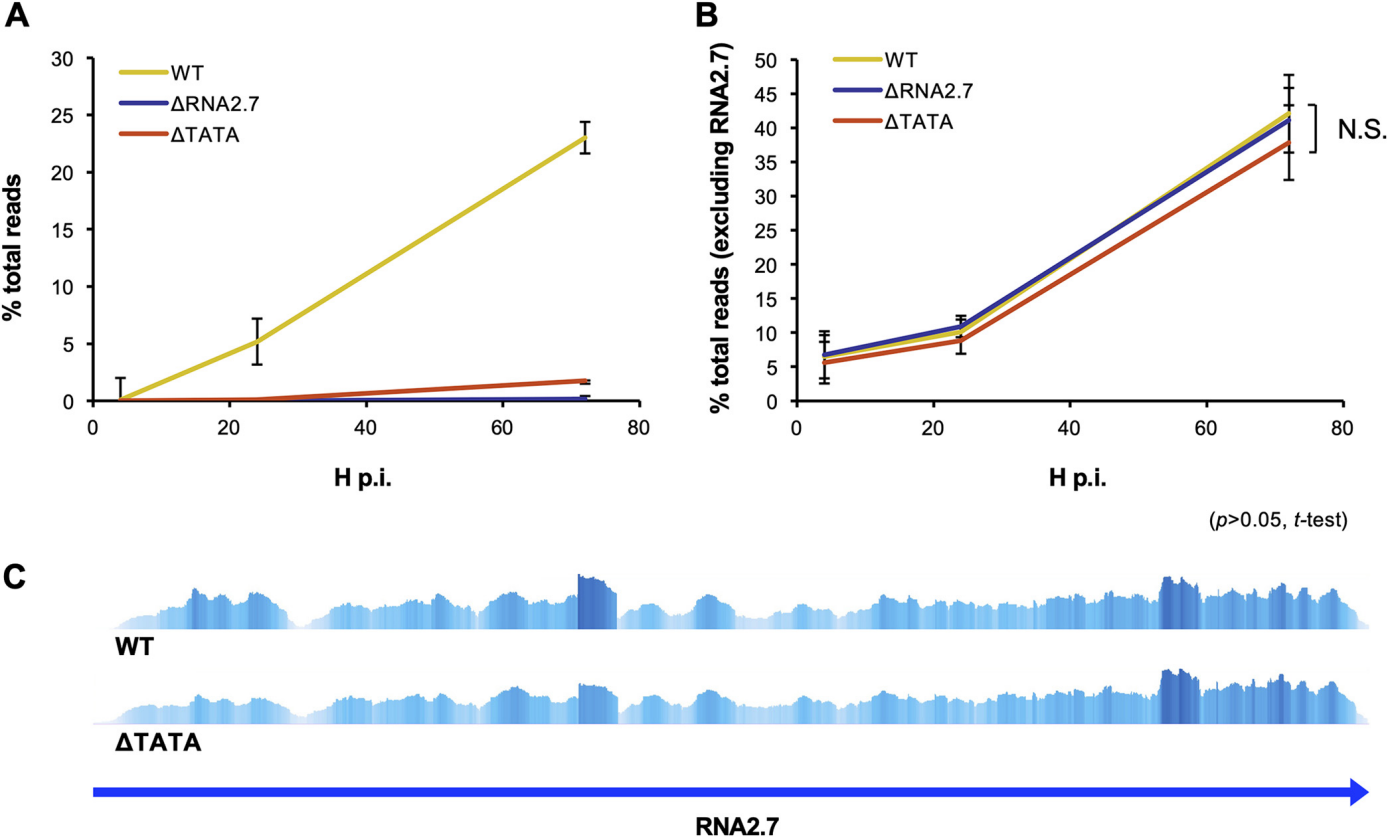
Effects of RNA2.7 on viral transcription. HFFF2 cells were infected at an MOI of  5 with WT, ΔRNA2.7, or ΔTATA. Sequence reads generated from polyadenylated RNA isolated at 4, 24, or 72 h p.i. were aligned to the WT and ΔRNA2.7 genome sequences and counted. Each error bar indicates the standard deviation of three independent experiments. The data are shown for reads from RNA2.7 (A) and all other viral transcripts (B) and are expressed as percentages of all viral and host reads (% total reads). N.S., not significant. (C) Alignment of sense reads from WT- and ΔTATA-infected cells at 72 h p.i. to the region containing the RNA2.7 gene, visualized in Tablet. Greater height and intensity and indicate higher coverage. The two plots are shown on different scales: ΔTATA expressed approximately 10% of the level of RNA2.7 expressed by WT (see panel A).

Previous observations that RNA2.7 is expressed with early kinetics and accumulates as infection progresses (18, 19) were confirmed (Fig. 2A). The levels of all other viral transcripts were the same for each virus (Fig. 2B; see also Table S1A in the supplemental material), indicating that the absence of RNA2.7 had no general effect on the proportion of viral RNA (excluding RNA2.7) expressed during the course of infection. These data dealt with the aggregate of viral RNAs and took no account of the behavior of individual RNAs or whether they were sense or antisense (i.e., transcribed in the same direction as a gene or the opposite direction) (22, 31–33). To analyze individual genes, the numbers of reads originating from the sense and antisense strands of each of the 170 protein-coding CDSs and four lncRNA-encoding regions were counted. Comparisons excluded RNA2.7, and differential regulation by RNA2.7 was registered when exhibited by both ΔRNA2.7 and ΔTATA above a defined probability threshold (*q *<* *0.05).

**FIG 3:**
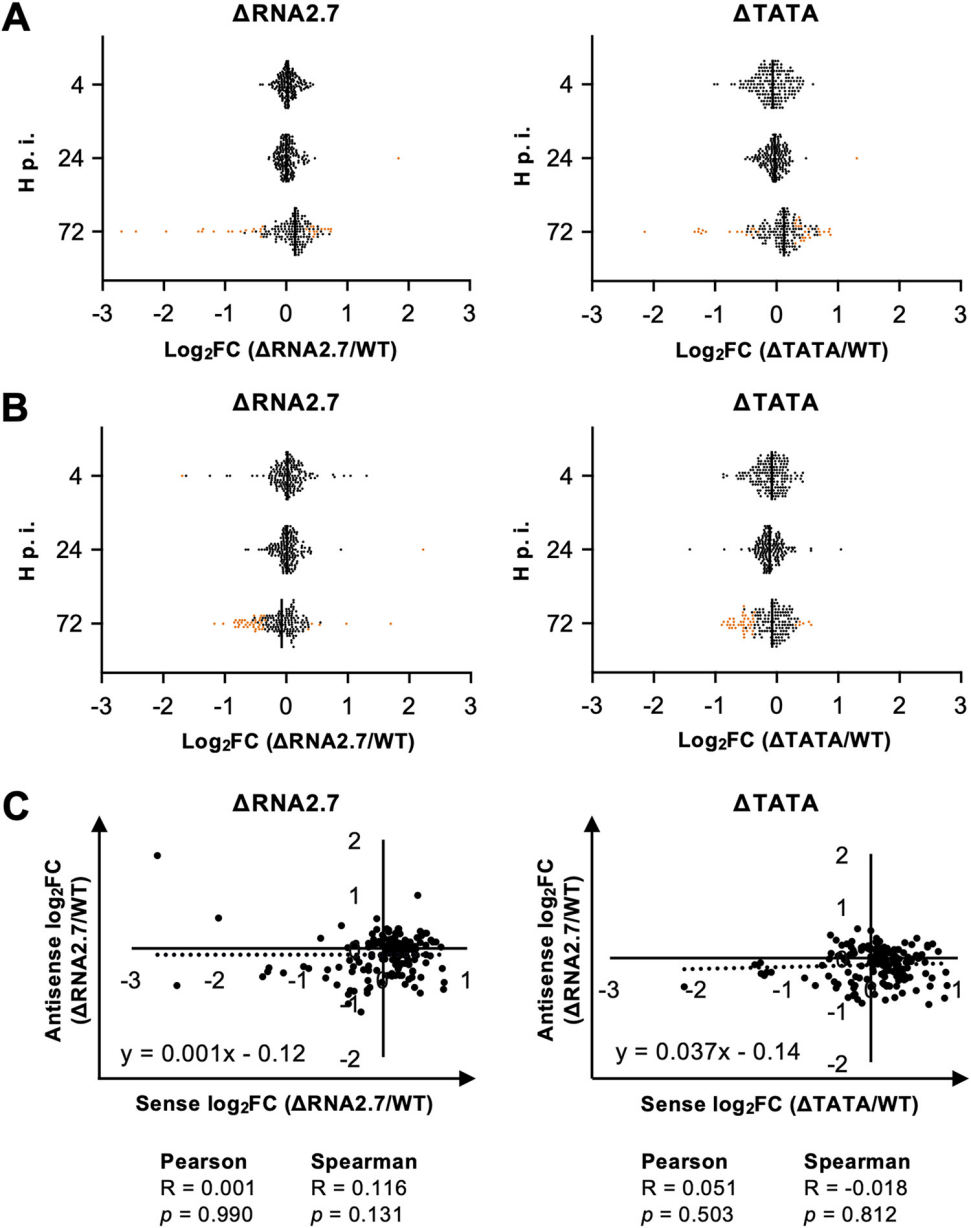
Effects of RNA2.7 on viral sense and antisense transcription. HFFF2 cells were infected at an MOI of  5 with WT, ΔRNA2.7, or ΔTATA. Sequence reads generated from polyadenylated RNA isolated at 4, 24, or 72 h p.i. were aligned to the WT and ΔRNA2.7 genome sequences, sorted into those originating from sense and antisense transcripts from each CDS, and counted. Sense (A) and antisense (B) expressions of viral transcripts by the mutants were compared to those of WT and are shown as log_2_fold change (log_2_FC) values. Significantly dysregulated transcripts (*q < *0.05) are marked by orange dots. (C) Lack of correlation between sense and antisense log_2_FC values in the RNA2.7 mutants. The data were generated from three independent experiments.

No sense transcripts were differentially expressed by the mutants at 4 h p.i., and only one gene (RL1) was upregulated at 24 h p.i. (Fig. 3A and Table 1, part A; see also Table S1B). In contrast, sense transcripts from 22 genes were differentially expressed at 72 h p.i., with nine upregulated and 13 downregulated (Table 1, part B). Differential expression by ΔRNA2.7 was moderate, ranging from upregulation by 1.66-fold to downregulation by 6.47-fold. RL5A, the 3′-untranslated region (3′ UTR) of which overlaps RNA2.7, was the third most downregulated gene (3.89-fold). Despite the lack of the TATA box, expression of RNA2.7 at 72 h p.i. was observed in ΔTATA at approximately 10% of the level in WT (Fig. 2A). The similarity of the transcription profiles of this region of the genome in WT and ΔTATA provided no evidence for an alternative promoter contributing significantly in the latter (Fig. 2C). No antisense transcripts were differentially regulated at 4 or 24 h p.i. At 72 h p.i., antisense transcripts to three genes in ΔRNA2.7-infected cells were upregulated by ≤1.45-fold, and transcripts antisense to 26 genes were downregulated by ≤2.25-fold (Fig. 3B and Table 1, part C; see also Table S1C). Only two loci were consistently dysregulated in both sense and antisense transcription (RL10 and UL142, both downregulated). There was no overall correlation between the differential expression of sense and antisense transcripts for all viral genes in mutant-infected cells at 72 h p.i. (Spearman’s correlation, *P > *0.05; Fig. 3C).

**TABLE 1:** Effects of RNA2.7 on viral transcription^*a*^

Part, time p.i., and ORF	Comparison				
	ΔRNA2.7 vs WT		ΔTATA vs WT		
	log_2_FC	*q*	ID	log_2_FC	*q*
Part A, 24 h					
RL1	1.84	0.001	RL1	1.31	0.036
					
		
Part B, 72 h					
US9	0.73	0.0160	US9	0.88	0.0175
US8	0.73	0.0153	US8	0.85	0.0183
UL57	0.69	0.0073	UL57	0.55	0.0164
UL37	0.63	0.0424	UL37	0.71	0.0338
UL56	0.57	0.0176	UL56	0.43	0.0459
UL130	0.46	0.0274	UL130	0.47	0.0439
UL72	0.45	0.0208	UL72	0.45	0.0299
UL20	0.45	0.0220	UL20	0.52	0.0299
UL55	0.45	0.0160	UL55	0.32	0.0420
US26	−0.42	0.0153	US26	−0.30	0.0447
US7	−0.55	0.0208	US7	−0.40	0.0439
UL10	−0.66	0.0208	UL10	−0.56	0.0408
UL144	−0.68	0.0094	UL144	−0.64	0.0139
UL145	−0.74	0.0073	UL145	−0.40	0.0420
RL10	−0.94	0.0056	RL10	−0.76	0.0089
UL8	−1.19	0.0073	UL8	−1.20	0.0057
UL9	−1.36	0.0073	UL9	−1.33	0.0052
UL7	−1.37	0.0040	UL7	−1.26	0.0052
UL1	−1.43	0.0028	UL1	−1.15	0.0052
RL5A	−1.96	0.0004	RL5A	−1.22	0.0052
UL142	−2.46	0.0220	UL142	−2.15	0.0408
RL6	−2.69	0.0004	RL6	−1.27	0.0068
					
		
Part C, 72 h					
UL124	0.54	0.0070	UL124	0.56	0.0172
UL73	0.41	0.0144	UL73	0.43	0.0172
UL32	0.36	0.0275	UL32	0.37	0.0237
RL10	−0.34	0.0459	RL10	−0.39	0.0470
RL12	−0.41	0.0452	RL12	−0.53	0.0238
UL23	−0.41	0.0254	UL23	−0.56	0.0130
UL91	−0.43	0.0305	UL91	−0.54	0.0463
US13	−0.43	0.0144	US13	−0.54	0.0115
US28	−0.44	0.0396	US28	−0.47	0.0402
UL16	−0.50	0.0111	UL16	−0.43	0.0238
UL72	−0.53	0.0081	UL72	−0.38	0.0320
US10	−0.53	0.0075	US10	−0.54	0.0149
US12	−0.57	0.0098	US12	−0.76	0.0046
UL6	−0.59	0.0029	UL6	−0.42	0.0226
US9	−0.63	0.0029	US9	−0.53	0.0091
UL71	−0.63	0.0469	UL71	−0.81	0.0172
US18	−0.66	0.0028	US18	−0.67	0.0043
US7	−0.67	0.0037	US7	−0.58	0.0098
UL142	−0.69	0.0111	UL142	−0.55	0.0238
US11	−0.72	0.0134	US11	−0.89	0.0091
US8	−0.76	0.0012	US8	−0.77	0.0043
UL5	−0.76	0.0028	UL5	−0.70	0.0046
US6	−0.78	0.0028	US6	−0.70	0.0043
RL13	−0.79	0.0029	RL13	−0.73	0.0091
RL9A	−0.82	0.0134	RL9A	−0.79	0.0226
UL20	−0.83	0.0037	UL20	−0.62	0.0172
US27	−0.85	0.0012	US27	−0.76	0.0043
US3	−1.03	0.0029	US3	−0.58	0.0172
UL2	−1.170	0.0009	UL2	−0.866	0.0046

a HFFF2 cells were infected at an MOI of 5 with WT, ΔRNA2.7, or ΔTATA. Strand-specific sequence reads were generated from polyadenylated RNA isolated at 24 or 72 h p.i., sorted into reads originating from the sense and antisense transcripts for each CDS, and counted. The results are shown as log_2_fold change (log_2_FC) values. Significantly dysregulated transcripts (*q < *0.05) are listed for sense (parts A and B) and antisense (part C) transcripts. The results of three independent experiments are shown.

No differentially expressed cellular RNAs were identified at 4 h and 24 h p.i. At 72 h p.i., 1,552 genes were differentially expressed in ΔRNA2.7-infected cells, and 1200 were differentially expressed in ΔTATA-infected cells (see Table S2). The differences in expression level were largely moderate: 99% of the genes dysregulated in ΔRNA2.7-infected cells and ΔTATA-infected cells were differentially expressed by ≤4-fold. In both mutants, most dysregulated genes (74 to 76%) were downregulated. Of the cellular genes differentially regulated by the mutants at 72 h p.i., 931 were common to both ΔRNA2.7 and ΔTATA, with 146 upregulated and 785 downregulated (see Table S2).

### Role of cell movement genes.

The functions of the 931 cellular genes dysregulated by the RNA2.7 mutants were investigated by pathway analysis (Table 2). The most prominent category was cell movement, into which 108 genes fell and which scored strongly by gene enrichment (*P = *6.83E–10, Fisher exact test) and the concerted up- or downregulation of these genes (*z* = −5.187). Many genes in other high-scoring categories, such as migration of cells and invasion of cells, were common to the cell movement category (final column in Table 2). Several of these cell-movement-associated (CMA) genes were investigated further.

**TABLE 2:** Functional categorization of transcripts differentially expressed by RNA2.7 mutants^*a*^

Function or effect	*P*	Predicted activation state in RNA2.7 mutants	*z* score	No. of genes	No. of genes in cell movement category
Cell movement	6.83E–10	Decreased	−5.187	108	108
Migration of cells	3.66E–08	Decreased	−4.688	95	95
Invasion of cells	3.28E–05	Decreased	−4.064	53	30
Migration of endothelial cells	1.21E–04	Decreased	−3.857	27	27
Invasion of tumor cell lines	4.48E–04	Decreased	−3.795	41	23
Cell movement of endothelial cells	7.57E–05	Decreased	−3.739	29	29
Homing of cells	1.20E–03	Decreased	−3.729	30	30
Development of vasculature	6.16E–03	Decreased	−3.705	38	0
Angiogenesis	9.11E–03	Decreased	−3.705	37	0
Chemotaxis	1.93E–03	Decreased	−3.607	29	29
Chemotaxis of cells	3.47E–03	Decreased	−3.607	27	27
Formation of cellular protrusions	1.07E–04	Decreased	−3.498	33	14
Cell movement of leukocytes	4.17E–03	Decreased	−3.430	29	29
Organization of cytoskeleton	1.52E–04	Decreased	−3.321	56	27
Cellular homeostasis	6.61E–05	Decreased	−3.295	63	0
Microtubule dynamics	1.79E–04	Decreased	−3.130	42	18
Proliferation of blood cells	6.18E–03	Decreased	−2.988	28	0
Invasion of carcinoma cell lines	1.23E–02	Decreased	−2.909	15	0
Endothelial cell development	1.61E–02	Decreased	−2.901	21	0

a Pathway analysis of the 931 regulated cellular genes. Significance is indicated by the *P* value, and increased or decreased activity of the implicated biological function by the *z* score.

**FIG 4:**
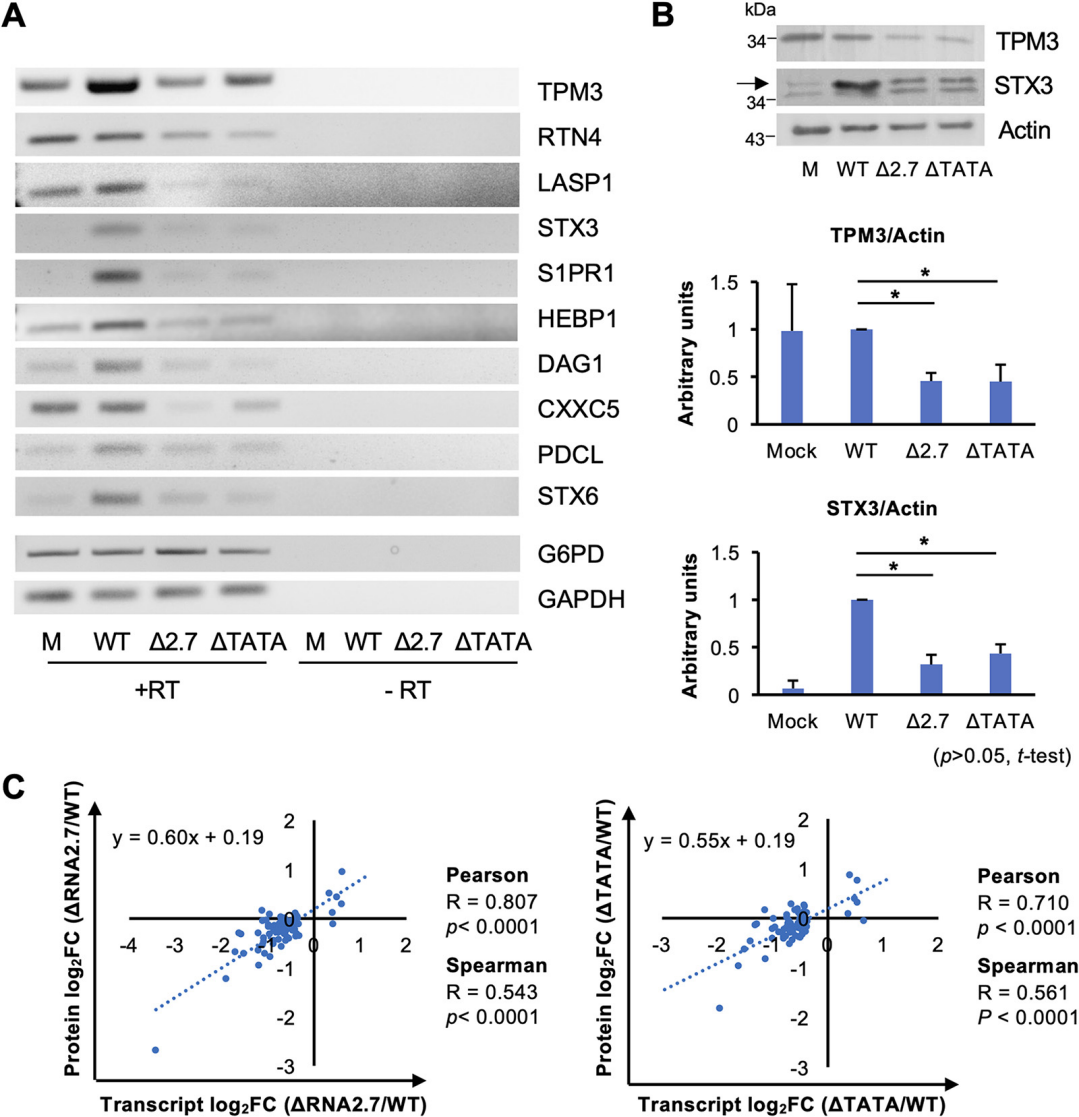
Effects of RNA2.7 on expression of regulated CMA genes. (A) Synchronized HFFF2 cells were infected at an MOI of  5 with WT, ΔRNA2.7 (denoted Δ2.7), or ΔTATA. At 72 h p.i., the transcript levels of 10 cell movement-associated (CMA) genes that were highly expressed in WT-infected cells and downregulated in mutant-infected cells in the transcriptomic analysis were confirmed by RT-PCR, with GAPDH and G6PD serving as loading controls. One representative result of three independent experiments is shown. (B) Protein levels for two of these genes examined by immunoblotting analysis. Band density was normalized to the actin loading control, and expression levels relative to WT-infected cells for three independent experiments are shown. (C) Differential expression data for the 73 regulated CMA genes that were registered in both the transcriptomic and proteomic analyses. These were calculated as log_2_fold change (log_2_FC) values relative to WT. R and *p* define the strength and significance of the correlation, respectively. M, mock infected. Error bars denote standard deviation values.

**FIG 5:**
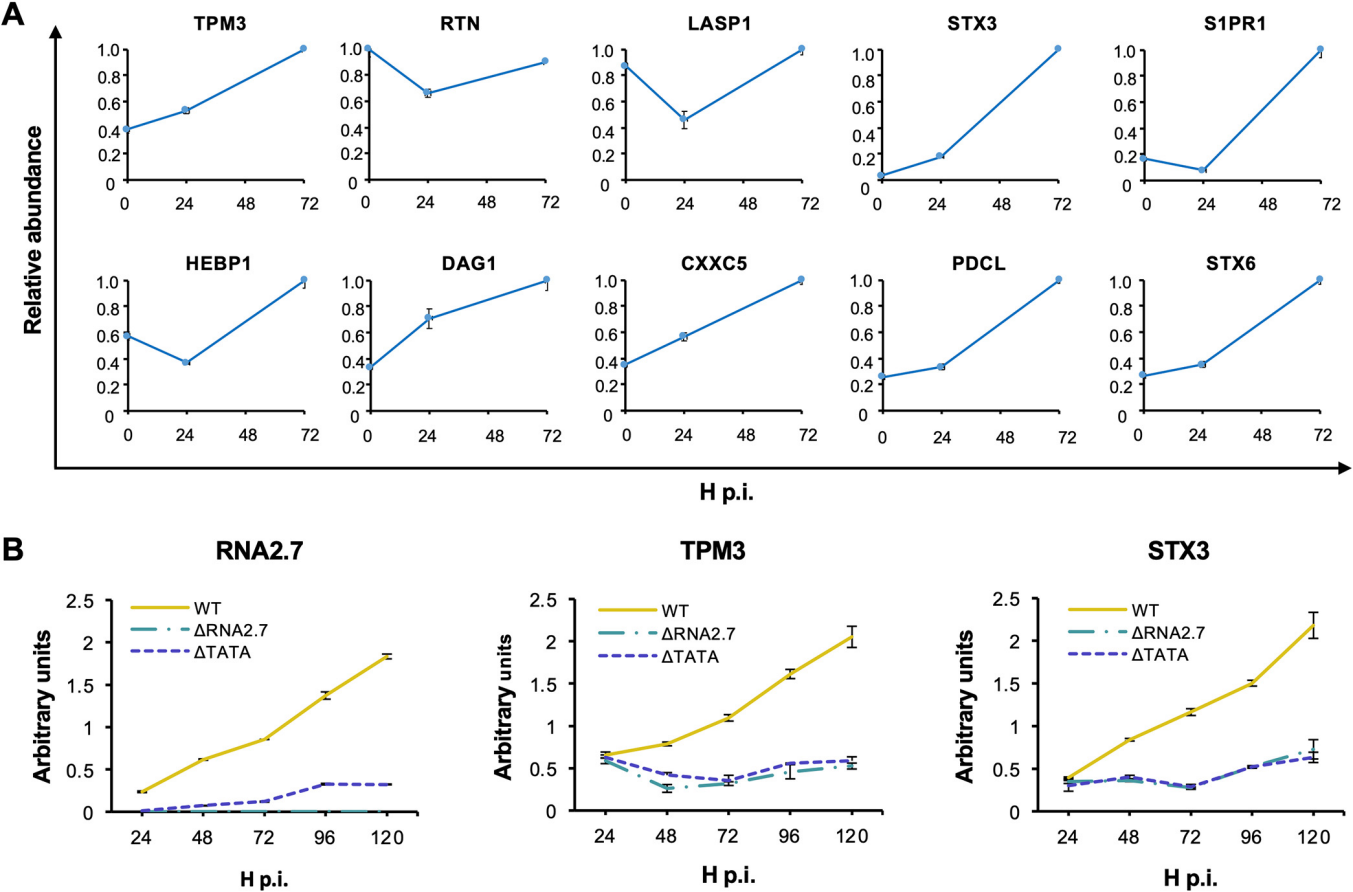
Effects of HCMV infection on transcription of CMA genes. HFFF2 cells were infected at an MOI of  5 with WT, ΔRNA2.7, or ΔTATA. (A) Data for 10 highly expressed CMA genes that were upregulated in the presence of RNA2.7 were extracted from Table S1 of a published report (36). Immortalized fibroblasts (HFFF-TERT cells) were infected at an MOI of 5 or 10 with HCMV strain Merlin in three biological replicates, and transcript abundance was determined by sequencing RNA samples harvested at 0 (mock infected), 24, or 72 h p.i. RPKM values were normalized for each gene to the highest level observed during the time course. (B) Data for RNA2.7, TPM3, and STX3 transcripts determined by RT-qPCR and normalized to that of the GAPDH transcript. Each result represents one of three independent experiments, with error bars indicating the standard deviation of technical replicates.

Of the 108 CMA genes differentially expressed by the RNA2.7 mutants, 99 were downregulated and nine were upregulated (see Table S3A). Ten genes were selected for further analysis that were highly expressed in WT-infected cells and dysregulated (all downregulated) by >1.8-fold in ΔRNA2.7-infected cells and by >1.6-fold in ΔTATA-infected cells (Table 3). These and all subsequent experiments were performed in HFFF2 cells that had been synchronized to the G_0_ phase of the cell cycle by serum starvation for 48 h prior to infection. Analysis of transcription of these genes by reverse transcription-PCR (RT-PCR) showed that transcript levels were either maintained or upregulated by infection of cells with WT and markedly decreased during infection with ΔRNA2.7 or ΔTATA (Fig. 4A). Previously published transcriptomic data (34) utilizing nonsynchronized cells showed that all 10 genes were upregulated in WT-infected cells between 24 and 72 h p.i. (Fig. 5A). Two of these genes, encoding Tropomyosin 3 (TPM3) and Syntaxin 3 (STX3), were investigated further. Transcripts accumulated for each between 24 and 120 h p.i. in WT-infected cells but remained at basal levels in cells infected with ΔRNA2.7 or ΔTATA (Fig. 5B), and immunoblotting analysis confirmed decreased protein levels of both proteins in cells infected with ΔRNA2.7 or ΔTATA (Fig. 4B). These experiments validated the downregulation of 10 of the 108 regulated CMA genes at the transcriptional level and two at the protein level. Preliminary evidence that CMA protein levels follow transcript levels more generally was obtained by a single proteomic analysis of WT, ΔRNA2.7 and ΔTATA-infected cells at 72 h p.i., in which 73 of the 108 CMA genes were assessed (see Table S4). Protein and transcript levels correlated strongly (Fig. 4C), although only the members of a subset of CMA proteins were significantly dysregulated by ΔRNA2.7 and ΔTATA (28/73 [38%] and 19/73 [26%], respectively (*P < *0.05)).

**TABLE 3:** Effects of RNA2.7 on expression of regulated CMA genes as determined by transcriptomic assay^*a*^

Gene	Transcript level (RPKM)			Fold change vs WT (log_2_FC)			
	WT	Δ2.7	ΔTATA	Δ2.7	*q*	ΔTATA	*q*
TPM3	551.2	178.6	225.2	−1.626	0.001	−1.291	0.001
RTN4	338	171.6	209.8	−0.978	0.001	−0.688	0.001
LASP1	174.3	58.3	69.4	−1.580	0.001	−1.329	0.001
STX3	79.6	24.4	27.8	−0.878	0.001	−0.692	0.001
S1PR1	70.5	17.2	20.6	−1.706	0.001	−1.518	0.001
HEBP1	61.6	26.8	31.5	−2.035	0.001	−1.775	0.001
DAG1	55.8	26.2	30	−1.201	0.001	−0.968	0.001
CXXC5	44.3	15.7	19.4	−1.091	0.001	−0.895	0.001
PDCL	43.6	19.1	19.8	−1.497	0.001	−1.191	0.001
STX6	29	13.7	14.3	−1.191	0.001	−1.139	0.001

a Transcriptomic analysis was performed at 72 h p.i. of HFFF2 cells infected at an MOI of 5 with WT, ΔRNA2.7 (denoted Δ2.7), or ΔTATA. The transcript levels of 10 CMA genes that are highly expressed in WT-infected cells and downregulated in RNA2.7 mutant-infected cells are shown as RPKM and log_2_fold change (log_2_FC) values. Significance is indicated by *q *<* *0.05.

**FIG 6:**
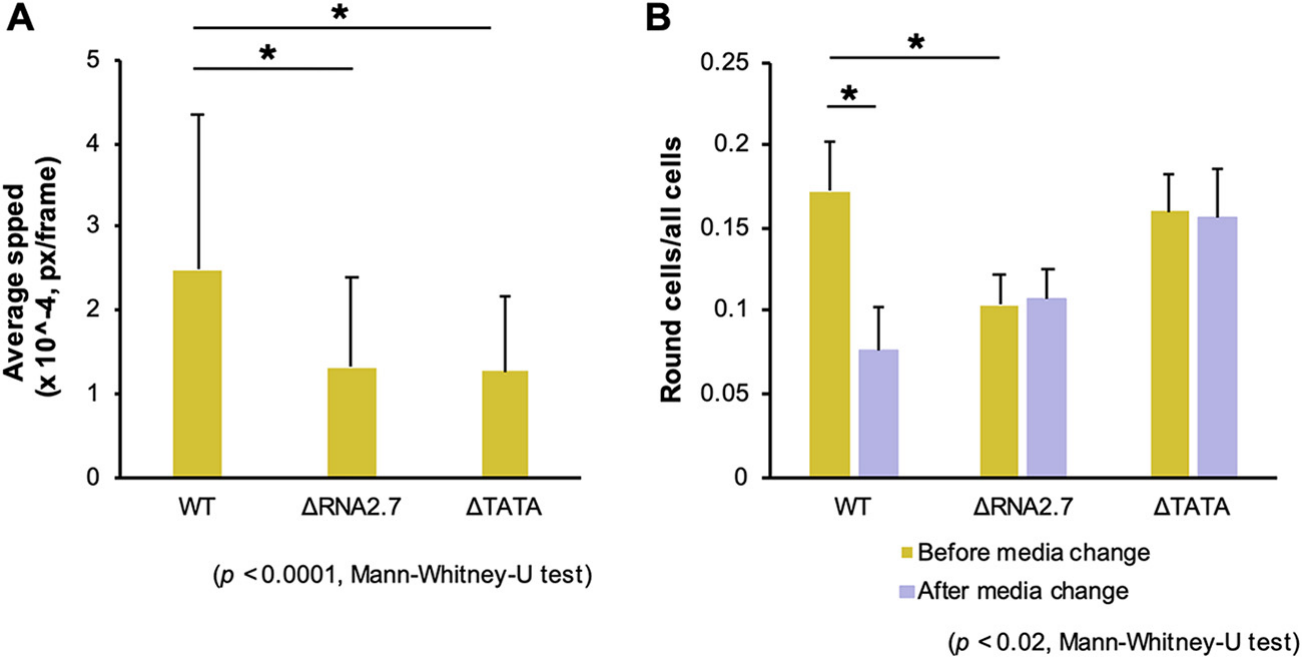
Effects of RNA2.7 on movement and rounding of cells. Synchronized HF-Tet cells were infected at an MOI of 5 with UL36-GFP-WT, UL36-GFP-ΔRNA2.7 (denoted ΔRNA2.7), or UL36-GFP-ΔTATA (denoted ΔTATA). (A) Fluorescent live cell microscopy and cell tracking performed at 72 to 120 h p.i., with images taken every 10 min. The data represent three independent experiments, each involving duplicate wells and five independent fields of view per well. An average of 756 good quality tracks per sample was analyzed. (B) Infected cell roundness, as defined by an aspect (breath versus width) ratio of ≥0.7 measured at 96 h p.i. before and after removing detached cells by changing the culture medium. The data were generated from three independent experiments. Px, pixels.

The results described above implied that cells infected with the RNA2.7 mutants would exhibit lower motility than WT-infected cells at late times in infection. To investigate this, the two RNA2.7 mutations were introduced into the Merlin genome with the green fluorescent protein (GFP) CDS fused to a P2A self-cleaving peptide at the 3′ end of the UL36-coding region, in order to identify infected cells by live-cell microscopy. Human fibroblasts expressing the tetracycline (Tet) repressor (HF-Tet) cells were infected with the parental virus (termed UL36-GFP-WT), UL36-GFP-ΔRNA2.7 or UL36-GFP-ΔTATA, and the speed of movement of infected cells was measured from 72 until 120 h p.i. The results showed that UL36-GFP-WT-infected cells moved 1.6 to 2.2 times faster than UL36-GFP-ΔRNA2.7- or UL36-GFP-ΔTATA-infected cells (Fig. 6A), thus supporting the conclusion that RNA2.7 is involved in promoting the movement of infected cells. Microscopic analysis also revealed that, although cells infected with the RNA2.7 mutants readily developed cytopathic effects, the number of cells that became spherical was lower by 40% in UL36-GFP-ΔRNA2.7-infected cells but not lower in UL36-GFP-ΔTATA-infected cells (Fig. 6B). Moreover, when infected spherical cells that had become detached from the monolayer were removed by replacing the medium, the number of remaining infected spherical cells was lower by 55% in UL36-GFP-WT-infected cells but not lower in mutant-infected cells. These data suggest that, although low-level expression of RNA2.7 by UL36-GFP-ΔTATA (7 to 14% of WT; Fig. 2A and Fig. 5B) was sufficient to promote rounding of infected cells, high-level expression was required for increased detachment. Relevant to this phenotype, 15 of the 931 transcripts dysregulated by the RNA2.7 mutants are associated with focal adhesion formation (see Table S3B). Overall, these results indicate that RNA2.7 may coordinate the regulation of CMA genes and thus enhance infected cell movement late in infection.

**FIG 7:**
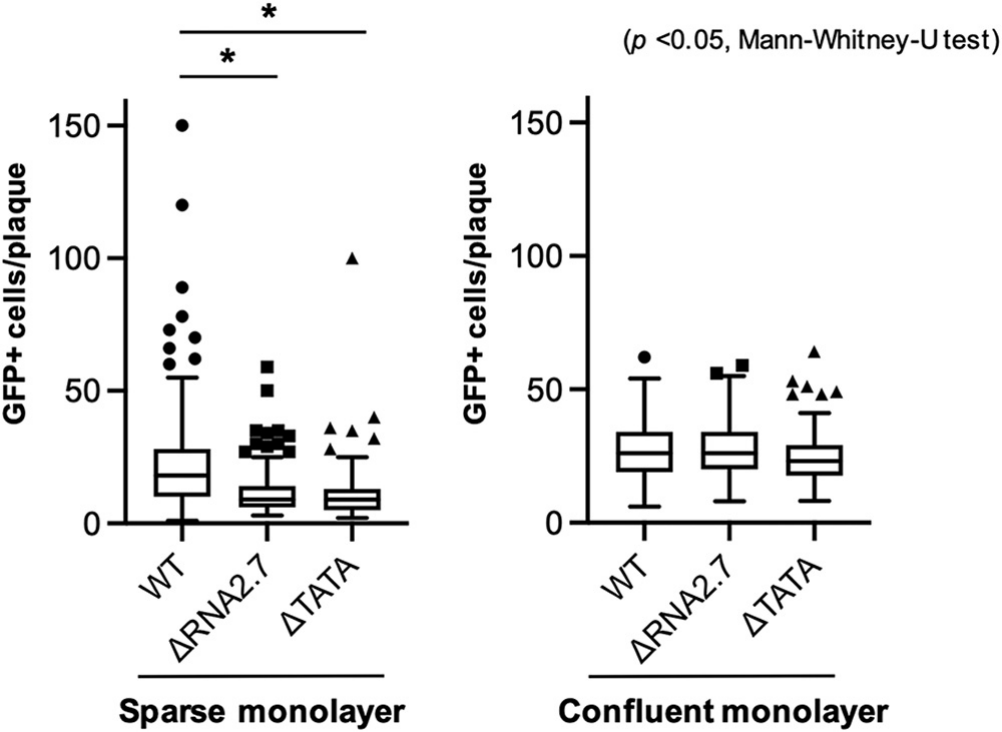
Effects of RNA2.7 on viral spread. Synchronized HF-Tet cells were infected at an MOI of 5 with UL36-GFP-WT, UL36-GFP-ΔRNA2.7 (denoted ΔRNA2.7), or UL36-GFP-ΔTATA (denoted ΔTATA). At 72 to 96 h p.i., infected cells were used to seed plaques on sparse or confluent monolayers in the presence of anti-HCMV blocking antibodies. GFP-expressing cells at 48 h p.i. were enumerated. A summary of results with Tukey box-and-whisker plots representing three independent experiments in which all plaques were assessed is shown.

During infection, motile infected cells are likely to make multiple contacts with uninfected cells, thus facilitating cell-to-cell transfer of virus. Therefore, we hypothesized that promotion of cell movement may be important for viral spread. To investigate this, HF-Tet cells were infected with UL36-GFP-WT, UL36-GFP-ΔRNA2.7, or UL36-GFP-ΔTATA. Late in infection, isolated infected cells were used to initiate infection in plaque assays on sparse or fully confluent monolayers in the presence of anti-HCMV blocking antibodies, and the number of GFP-positive cells per plaque was monitored. In sparse monolayers, there were 1.9- to 2.0-fold more infected cells in plaques seeded by UL36-GFP-WT-infected cells than in those seeded by cells infected with either mutant, whereas there were no differences in confluent cell monolayers (Fig. 7). This again supports the conclusion that RNA2.7 may promote cell movement, resulting in increased cell-to-cell spread of infectious virus late in infection.

### Role of GRIM-19.

**FIG 8:**
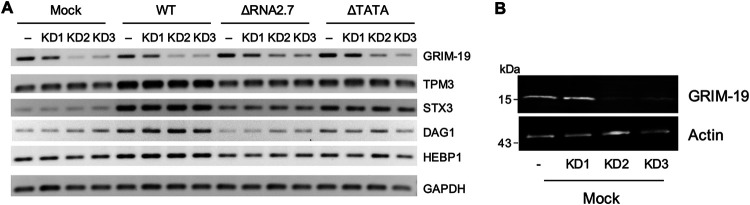
Effects of GRIM-19 on expression of regulated CMA genes. HFT cells were transduced with lentivirus constructs expressing either a nontargeting shRNA as negative control (−) or one of three separate GRIM-19-targeting shRNAs (KD1-KD3). Stable cell lines established by puromycin selection were synchronized and mock-infected (mock) or infected at an MOI of 5 with WT, ΔRNA2.7, or ΔTATA. (A) RT-PCR of CMA transcripts in RNA harvested at 72 h p.i., using GAPDH as a loading control. (B) Immunoblot of GRIM-19 levels in mock-infected cells. In each panel, the data represent one of three independent experiments.

The analyses described above implicate RNA2.7 in coordinating the regulation (mostly upregulation) of multiple cellular genes, presumably in order to achieve specific functional goals, such as increasing infected cell movement and viral spread. It is not clear how this is accomplished, but a common mechanism may be involved. One possibility is through interactions with GRIM-19, to which RNA2.7 binds directly (25). To investigate this, three independent shRNA knockdown cell lines (KD1-KD3) against GRIM-19, and a control cell line, were generated from human fibroblasts immortalized by lentiviral transduction of a telomerase gene (HFT cells). The effectiveness of knockdown was confirmed at the transcript (Fig. 8A, left 4 lanes) and protein levels (Fig. 8B), although that of KD1 was marginal. Expression of CMA genes during infection of these cell lines was also examined (Fig. 8A, right 12 lanes), and demonstrated that reduction in GRIM-19 level did not influence the level of transcription of any of the four CMA genes tested (TPM3, STX3, Dystroglycan 1 [DAG1], and Heme-binding protein 1 [HEBP1]). These data indicate that GRIM-19 is unlikely to be involved in the regulation of CMA genes involving RNA2.7.

### Role of viral genes.

Attempts were made using a lentivirus-based system to determine whether expression of RNA2.7 in the absence of other HCMV genes is sufficient to upregulate CMA genes. However, even though RNA2.7 was under the control of the powerful HCMV MIEP lacking the IE2-specific repression signal that is commonly used in expression systems, levels of RNA2.7 expression were <4% of those observed during HCMV infection. Efforts to rescue the RNA2.7 phenotype in the context of ΔRNA2.7 and ΔTATA infection were also unsuccessful, probably for the same reason. We also attempted using a recombinant adenovirus system at exceptionally high multiplicity of infection (MOI) to maximize RNA2.7 expression (also driven by the MIEP lacking the IE2-specific repression signal), but this resulted in the development of profound cytopathic effects. Infection with adenovirus under these conditions also prevented subsequent infection with HCMV, thus prohibiting complementation. These two widely used expression systems did not come anywhere near matching the power of the RNA2.7 promoter, and it was therefore not possible to assess the role of RNA2.7 in isolation or in complementing the RNA2.7 mutants.

**FIG 9:**
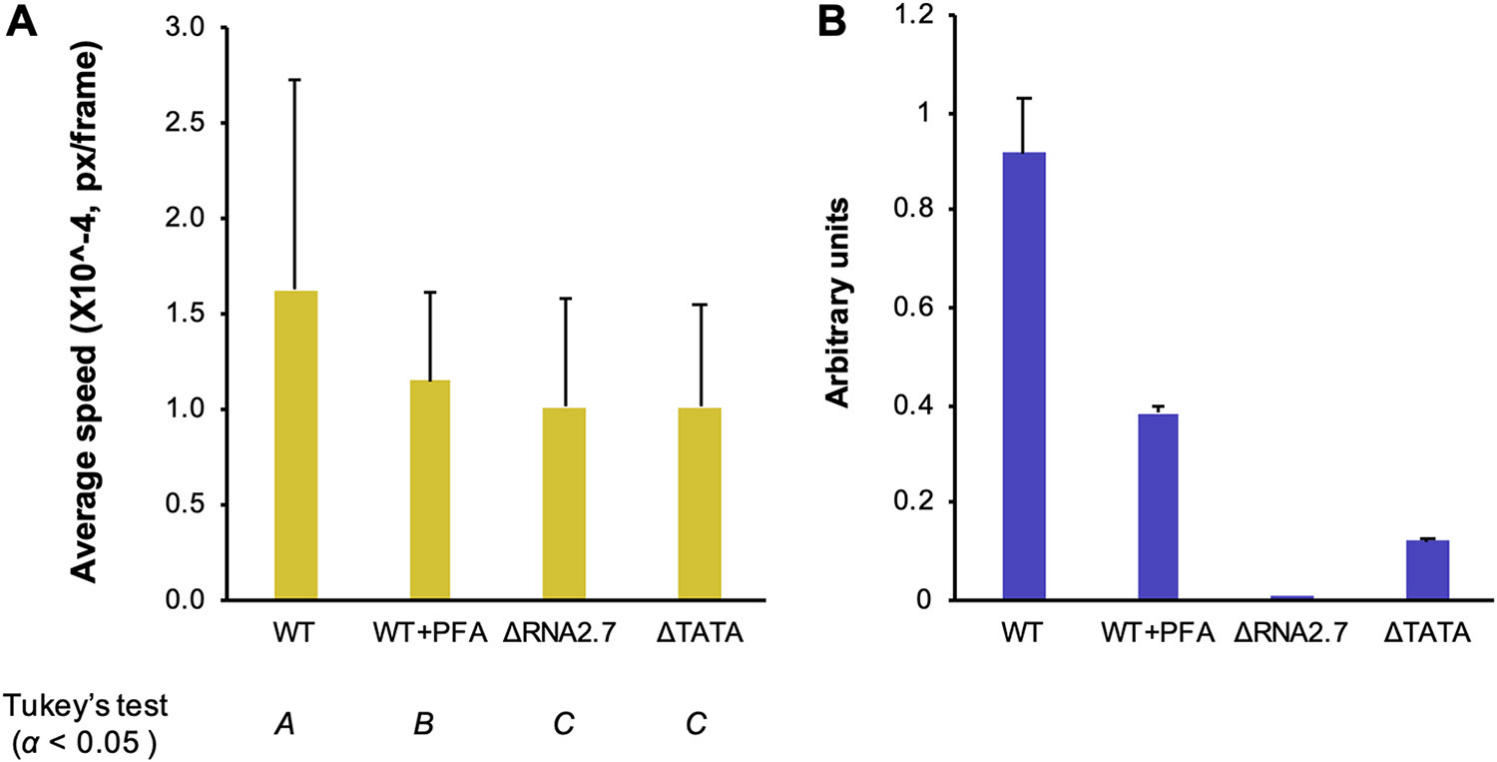
Effects of inhibiting viral DNA synthesis on cell movement. Synchronized HF-Tet cells were infected at an MOI of 5 with UL36-GFP-WT, UL36-GFP-ΔRNA2.7 (denoted ΔRNA2.7), or UL36-GFP-ΔTATA (denoted ΔTATA), after which the UL36-GFP-WT-infected cells were treated immediately with PFA or left untreated. (A) Cell movement speed measured by fluorescent live cell confocal microscopy and cell tracking at 48 to 96 h p.i., taking images at 10-min intervals. The data represent three independent experiments, each with duplicate wells and five independent fields of view per well. Significantly different results were grouped using Tukey’s test (columns *A*, *B*, and *C*), and error bars denote standard deviation values. (B) RNA2.7 levels measured by RT-qPCR at 72 h p.i., with GAPDH serving as a loading control. Error bars indicate standard deviation values between two independent experiments.

In an alternative approach to assessing whether regulation of CMA genes depends on the expression of other viral genes late in infection, phosphonoformic acid (PFA) was used to inhibit viral DNA synthesis and hence late gene expression. Cells infected with UL36-GFP-WT and treated with PFA moved 29% more slowly than untreated cells at 48 to 96 h p.i. (Fig. 9A), whereas cells infected with UL36-GFP-ΔRNA2.7 and UL36-GFP-ΔTATA moved 38% more slowly. These results suggest that expression of additional late viral genes is required for the regulation of CMA genes. However, although RNA2.7 is expressed with early kinetics, its level in treated cells was half that in untreated cells at 72 h p.i., raising the perhaps unlikely possibility that this lower level was insufficient to promote RNA2.7-mediated regulation (Fig. 9B).

### Role of RNA2.7 subdomains.

**FIG 10:**
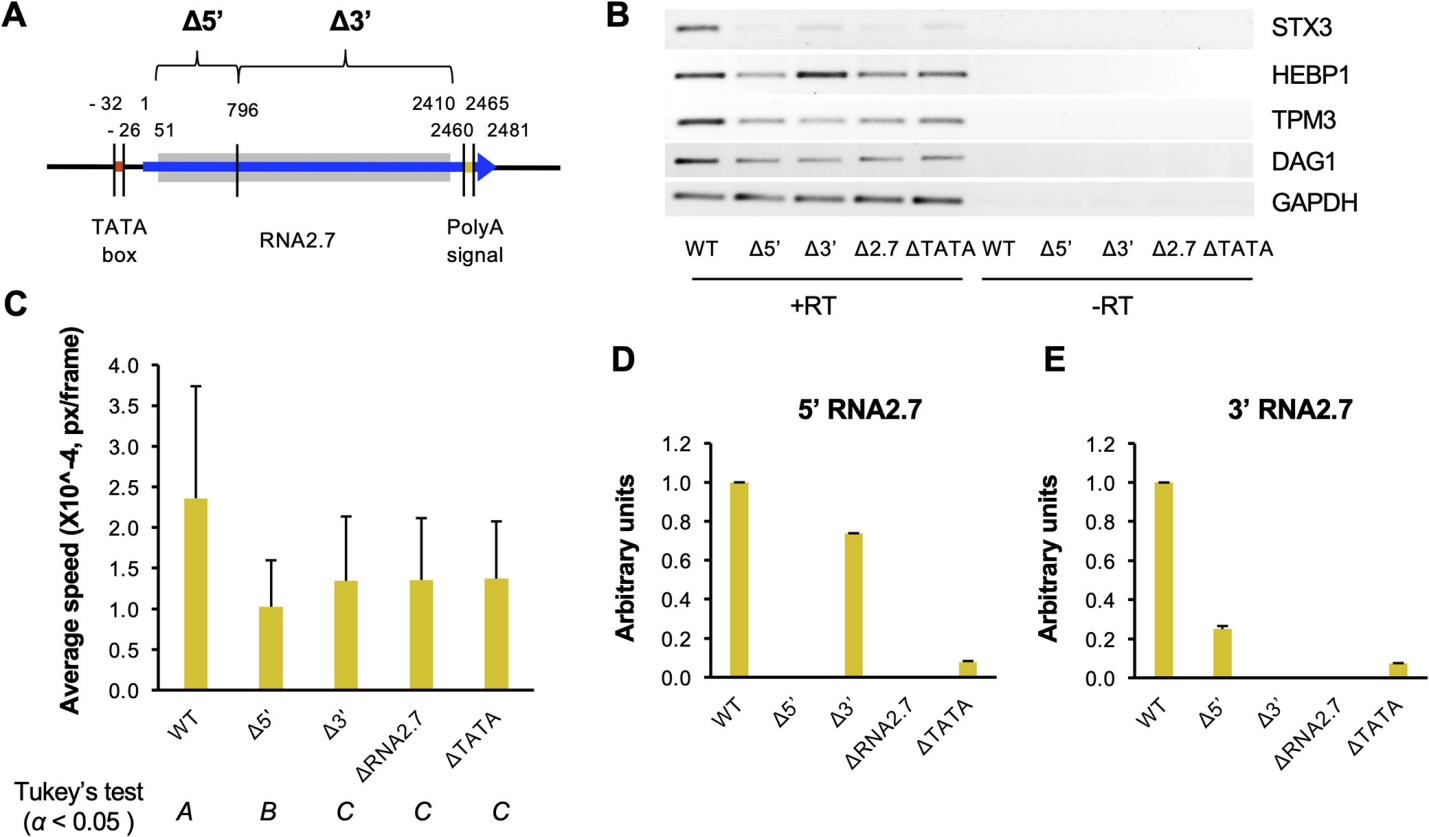
Effects of partially deleting RNA2.7 on expression of CMA genes and cell movement. (A) Diagram depicting the regions deleted in the RNA2.7 mutants (Δ5′ and Δ3′) in comparison to WT. The RNA2.7-coding region is indicated by a blue arrow, the TATA box by a red line, and the polyadenylation [poly(A)] signal by a yellow line. The deleted regions are marked by gray-shaded rectangles. Numbers indicate the distance (nt) from the transcription initiation site (at 1 nt). (B) HFFF2 cells were infected at an MOI of 5 with WT, Δ5′, Δ3′, ΔRNA2.7, or ΔTATA and CMA transcript levels in RNA harvested at 72 h p.i. were measured by RT-PCR, using GAPDH as a loading control. The data represent one of three independent experiments. RT, reverse transcriptase. (C) HF-Tet cells were infected at an MOI of 5 with UL36-GFP-WT, UL36-GFP-Δ5′ (denoted Δ5′), UL36-GFP-Δ3′ (denoted Δ3′), UL36-GFP-ΔRNA2.7 (denoted ΔRNA2.7), or UL36-GFP-ΔTATA (denoted ΔTATA). Cell movement at 72 to 120 h p.i. was measured by live cell confocal microscopy and tracking analysis. For each experiment, 10 random views of the field were analyzed per sample, resulting in the analysis of >1,700 tracks after excluding low quality tracks. Error bars denote standard deviation values. Significantly different results were grouped using Tukey’s test (columns *A*, *B*, and *C*). (D and E) Expression of the 5′ region (D) and the 3′ region (E) of RNA2.7 determined by RT-qPCR, normalizing levels to GAPDH. In each panel, the data represent three independent experiments, with error bars indicating standard deviation values.

Previously published work has shown that the 5′-terminal one-third of RNA2.7 (796 nt) is sufficient to bind to GRIM-19 and protect cells from apoptosis (27). To determine which parts of RNA2.7 are required for upregulation of CMA genes, mutants were generated that lacked either this subdomain or the remaining two-thirds of RNA2.7 (Δ5′ and Δ3′, respectively; Fig. 10A). The deletion in Δ5′ started 50 nt downstream of the transcription initiation site in order to limit promoter-associated effects on expression of the 3′ subdomain of RNA2.7, and the deletion in Δ3′ stopped 50 nt upstream of the polyadenylation signal in order to avoid affecting expression of the upstream gene RL5A, which shares this signal with RNA2.7.

The impact of the deletions on CMA gene expression during infection of HFFF2 cells was assessed. The levels of all four transcripts examined (TPM3, STX3, DAG1, and HEBP1) were reduced in Δ5′-infected cells to levels similar to those in ΔRNA2.7-infected cells. The same observation was made for two of these genes (TPM3 and DAG1) in Δ3′-infected cells, whereas the other two genes (STX3 and HEBP1) were expressed at greater levels than in ΔRNA2.7-infected cells (Fig. 10B). In addition, both UL36-GFP-Δ5′- and UL36-GFP-Δ3′-infected cells moved more slowly than UL36-GFP-WT-infected cells. The motility of UL36-GFP-Δ5′-infected cells and UL36-GFP-Δ3′-infected cells was 43 to 56% lower than that of WT-infected cells, and similar to that of UL36-GFP-ΔRNA2.7-infected cells and UL36-GFP-ΔTATA-infected cells, for which motility was decreased by 42 to 43% (Fig. 10C). In interpreting these results, it is important to note that the level of the residual part of RNA2.7 expressed in Δ3′-infected cells was similar to that of WT (74%), whereas the level in Δ5′-infected cells was only 25% and thus closer to that of ΔTATA (7%) than WT (Fig. 10D and E). As a result, it may be concluded that the 5′-subdomain cannot replace RNA2.7 in regulating CMA genes but that the role of the 3′-subdomain is unresolved.

### Role of mRNA stability.

**FIG 11:**
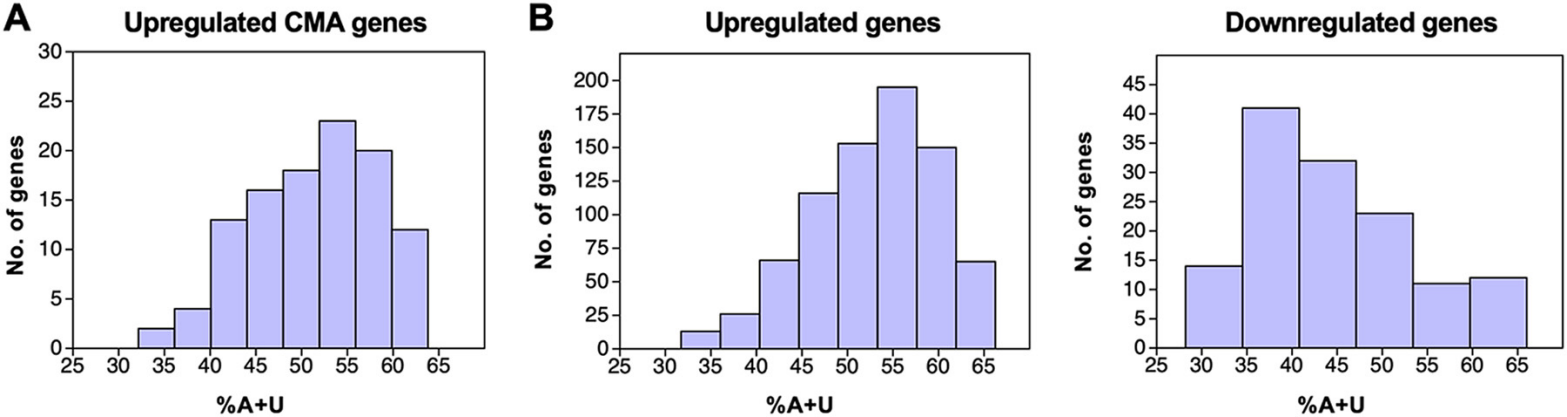
A+U content of mRNAs regulated in association with RNA2.7. (A) The 99 upregulated CMA mRNAs. (B) All 785 upregulated mRNAs and all 146 downregulated mRNAs.

The sequences of the 99 upregulated CMA mRNAs were analyzed for characteristics distinct from those of other cellular mRNAs. The majority (64 mRNAs) have an A+U content of >49.6%, which is greater than the average calculated for all cellular mRNAs (Fig. 11A). Since mRNA instability is associated with higher A+U content (35–41), we investigated whether CMA genes contain motifs associated with lower mRNA stability (35, 42, 43). These motifs comprise A+U-rich elements (ARE motifs 1 to 2E in Table 4) that are based on a pentamer AUUUA core sequence and are known to promote mRNA decay when present in the 3′ UTR (43–45). Other A+U-rich motifs (MEG, H1 to H3, and B1 to B4) and some G+C-rich motifs are associated with lower stability when present in specific regions of an mRNA, conversely, the presence of some G+C-rich motifs (H-1, H-2, B-1, and B-2) correlates with higher mRNA stability in certain instances (35).

**TABLE 4:** Effects of nucleotide composition and stability-associated motifs on mRNAs regulated in association with RNA2.7^*a*^

Feature or motif	Motif sequence	Upregulated CMA genes				Upregulated genes				Downregulated genes			
		5′ UTR	CDS	3′ UTR	mRNA	5′ UTR	CDS	3′ UTR	mRNA	5′ UTR	CDS	3′ UTR	mRNA
%A+U	–	R	–	–	E	R	E	E	E	R	R	R	R
1	[AU]AUUUA[AU]	–	–	**E**	**E**	–	**E**	**E**	**E**	–	**R**	**R**	**R**
2A	AUUUAUUUAUUUAUUUAUUUA	–	–	–	–	–	–	–	–	–	–	–	–
2B	AUUUAUUUAUUUAUUUA	–	–	–	–	–	–	–	–	–	–	–	–
2C	[AU]AUUUAUUUAUUUA[AU]	–	–	–	–	–	–	–	–	E	–	–	–
2D	[AU](2) AUUUAUUUA[AU](2)	–	–	–	–	–	–	**E**	E	E	–	–	–
2E	[AU](4) AUUUA[AU](4)	–	–	–	–	–	–	–	–	–	–	–	–
MEG	UUAUUUAUU	–	–	–	–	–	–	**E**	**E**	–	–	–	–
MEGSHORT	UAUUUAU	–	–	**E**	**E**	–	**E**	**E**	**E**	–	**R**	–	–
H1	UUUUUUU	**E**	–	**E**	**E**	–	–	**E**	**E**	–	–	**R**	**R**
H2	UUUUUAAA	–	–	**E**	**E**	–	–	**E**	**E**	–	–	**R**	**R**
H3	UUGUAAAUA	–	–	**E**	**E**	–	–	**E**	**E**	–	–	–	–
B1	UUUUAAAU	–	–	**E**	**E**	–	–	**E**	**E**	–	–	**R**	**R**
B2	UUUUAAUUU	*E*	–	–	–	–	–	**E**	**E**	–	–	–	–
B3	AAAUAUUUU	–	–	–	–	–	–	**E**	**E**	–	–	**R**	**R**
B4	AAUAUUUUU	–	–	–	–	–	–	**E**	**E**	–	–	–	–
H-1	CCGCCUC	E	–	–	–	E	–	–	**E**	–	**E**	–	–
H-2	CCAGCCUC	–	–	–	–	–	–	–	–	–	**E**	–	–
B-1	GGGCCUGG	–	–	–	–	–	R	–	–	*E*	E	E	*E*
B-2	CCCAGCCCC	–	–	–	–	–	–	–	–	–	–	*E*	E

a Occurrence per regulated mRNA of each sequence motif associated with stability or instability in comparison with an equal number of other human transcripts in 1,000 randomizations. Higher occurrences in regulated mRNAs in >950 comparisons (i.e., *P *<* *0.05) were considered to be enriched (E), and lower occurrences in >950 comparisons were considered to be reduced (R). Motifs enriched in mRNA regions associated with decreased or increased mRNA stability are indicated in boldface or italics, respectively. The results are shown for the 99 upregulated CMA mRNAs, all 785 upregulated mRNAs and all 146 downregulated mRNAs. [AU], A or U; numbers in parentheses following [AU] indicates the numbers of such nucleotides when >1. –, motif not enriched or reduced.

The occurrence of instability motifs in the 99 upregulated CMA mRNAs was compared to that in an equal number of randomly selected mRNAs that are not in this category, with this process being repeated 1,000 times. The A+U content of CMA mRNAs was higher than the randomly selected mRNAs in the whole mRNA and the 3′ UTR but lower in the 5′-untranslated region (5′ UTR; Table 4; see also Table S5A). These mRNAs were also enriched in six A+U-rich motifs located in regions of the mRNA associated with faster decay, including the ARE motif in the 3′ UTR. These results suggest that upregulated CMA genes tend to encode less-stable mRNAs and that RNA2.7 may upregulate them by reducing their rate of decay.

**FIG 12:**
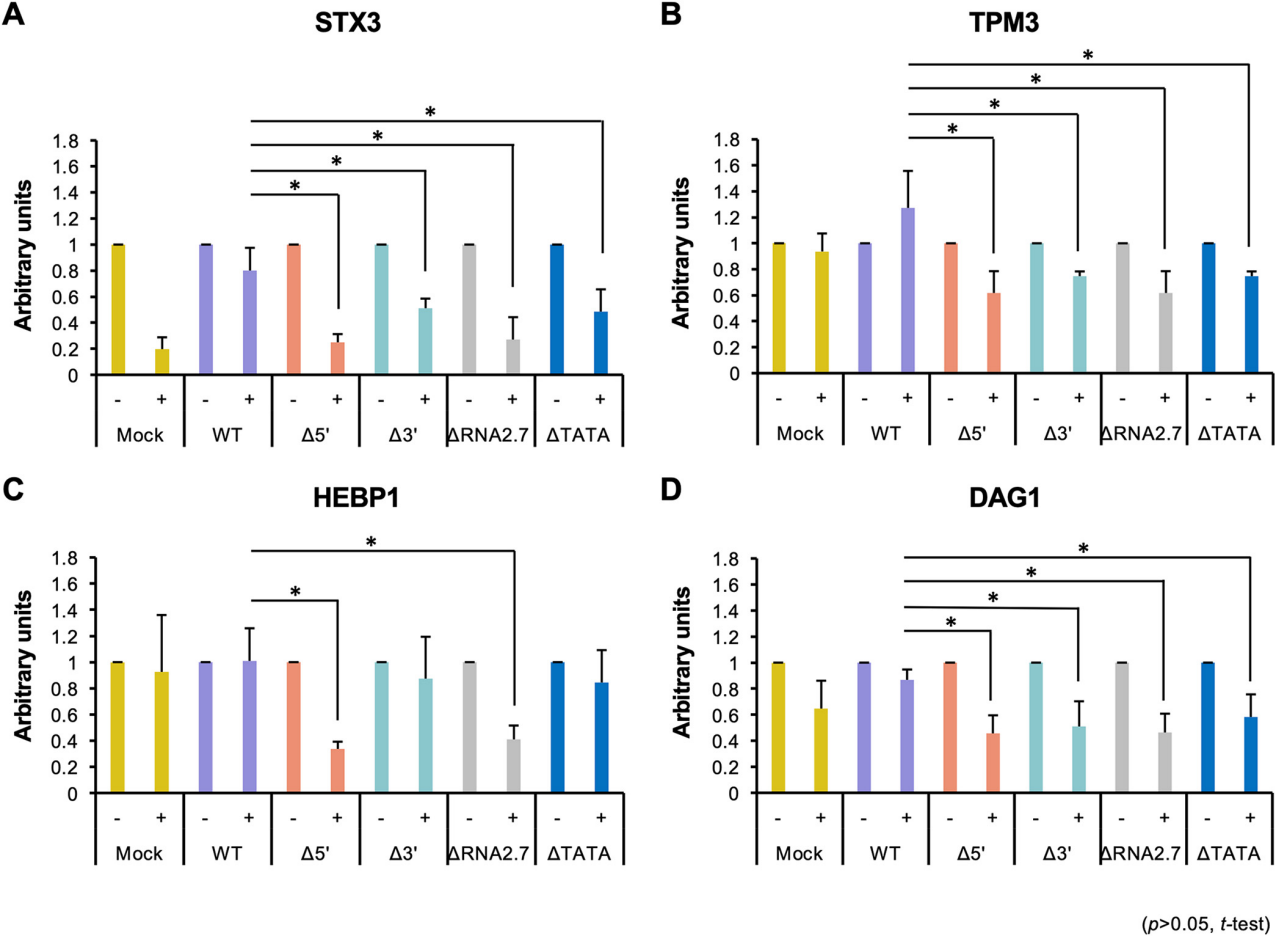
Effects of inhibiting RNA synthesis on persistence of regulated CMA transcripts. HFFF2 cells were infected at an MOI of 5 with WT, Δ5′, Δ3′, ΔRNA2.7 or ΔTATA, and then 12.5 μg/ml actinomycin D (+) or solvent only (−) was added at 72 h p.i. At 120 h p.i., CMA transcript levels were measured by RT-qPCR, normalizing levels to GAPDH: STX3 (A), TPM3 (B), HEBP1 (C), and DAG1 (D). The NT level for each virus was then normalized to 1, and the level in actinomycin D-treated samples was calculated relative to the relevant NT sample. Error bars indicate standard deviation values in four independent experiments.

To investigate this further, transcription in infected HFFF2 cells was blocked from 72 h p.i. by using actinomycin D to inhibit RNA polymerase II, so that mRNA levels thereafter would be affected by decay but not synthesis. The transcript levels of CMA genes were normalized to that of GAPDH, which encodes a stable transcript having an A+U content of 41 to 45% (depending on transcript variant) that has been used for this purpose in similar studies (46–48). For three of the CMA genes analyzed (STX3, TPM3, and DAG1), the proportion of transcript that persisted in the presence of actinomycin D was consistently higher in cells infected with WT than in cells infected with ΔRNA2.7, ΔTATA, Δ5′, or Δ3′ (Fig. 12A, B, and D). For HEBP1, the same trend was observed with ΔRNA2.7- and Δ5′-infected cells, but there was no significant difference between the persistence of transcripts in WT-infected cells and ΔTATA- or Δ3′-infected cells (Fig. 12C). These results indicate that RNA2.7 may upregulate CMA genes by inhibiting their decay.

To investigate whether the modulation of CMA mRNA stability involving RNA2.7 extends to other cellular mRNAs, the features associated with mRNA stability were collated for the 931 regulated genes (785 upregulated and 146 downregulated). As for the upregulated CMA mRNAs, the majority (69.3%) of all upregulated mRNAs had an A+U content of >49.6% (average, 52.8%; Fig. 11B). Moreover, when the upregulated mRNAs were compared to an equal number of randomly selected mRNAs that are not in this category, with a total of 1,000 comparisons, they had a higher overall A+U content in all comparisons (Table 4; see also Table S5A). These mRNAs were also enriched in motifs associated with lower stability, even more so than the 99 upregulated CMA mRNAs. The general pattern was one of higher A+U content in the CDS and 3′ UTR and a significant enrichment of A+U-rich decay-associated motifs (Table 4; see also Table S5A).

In contrast to the upregulated mRNAs, the majority (75.1%) of the 146 downregulated mRNAs had an A+U content of <49.6% (average, 44.5%; Fig. 11B), and the A+U content was significantly lower in all regions of the mRNA in 1,000 comparisons with other cellular mRNAs (Table 4; see also Table S5A). Also, the downregulated mRNAs were not enriched in A+U-rich decay-associated motifs, but instead were significantly reduced in six of them while having a higher than average occurrence of two stability-associated G+C-rich motifs (Table 4; see also Table S5A). In addition, two G+C-rich motifs associated with mRNA instability were present in the CDSs of downregulated mRNAs at above average frequency, although this may reflect the general G+C richness of these mRNAs. In summary, these findings indicate that nucleotide composition and the presence of motifs affecting RNA stability are prevailing features of the regulated cellular mRNAs, with upregulated transcripts typically comprising less stable A+U-rich mRNAs and downregulated transcripts comprising more stable G+C-rich mRNAs. In addition, although upregulated and downregulated mRNAs possess these opposing features, they share the common characteristic of being relatively G+C-rich in the 5′ UTR compared to other mRNAs.

**FIG 13:**
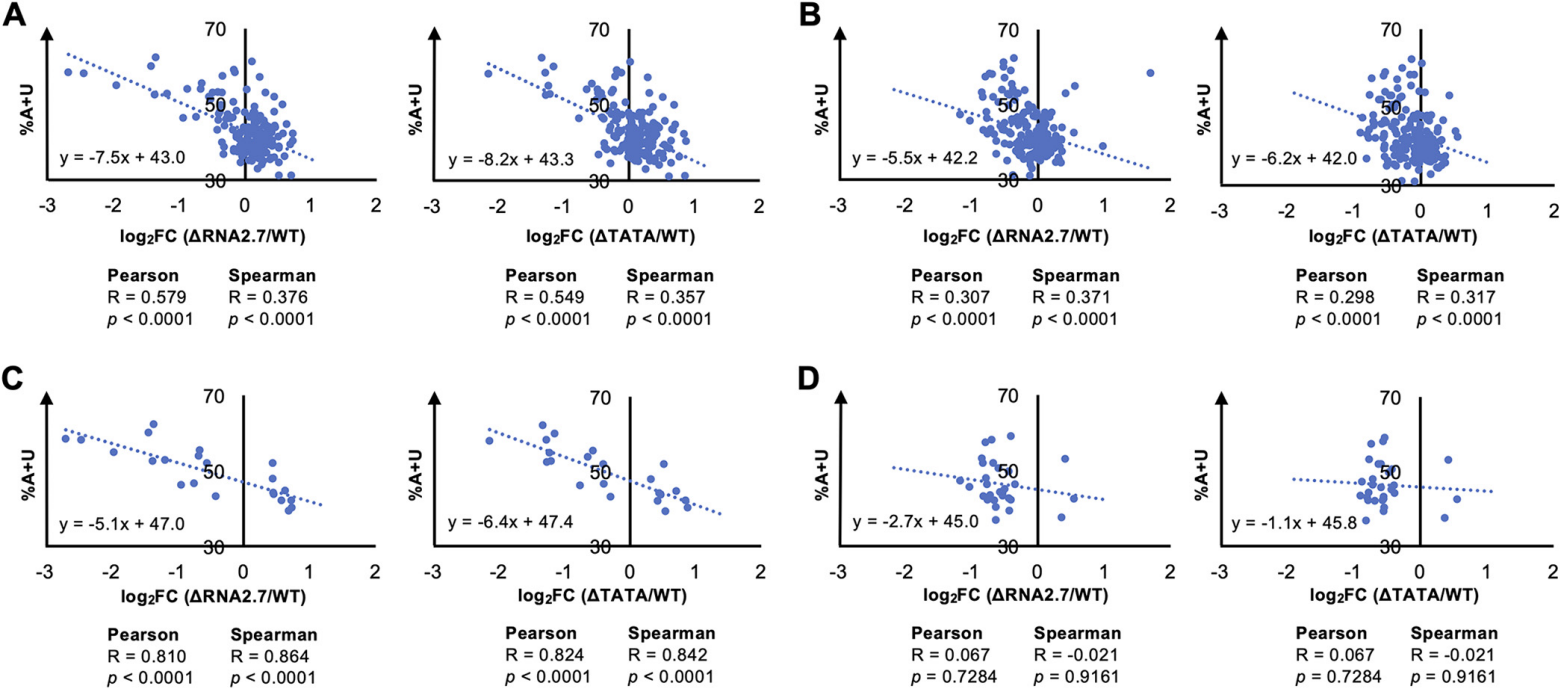
Effects of nucleotide composition on viral transcripts regulated in association with RNA2.7. HFFF2 cells were infected at an MOI of 5 with WT, ΔRNA2.7, or ΔTATA. Sequence reads generated from polyadenylated RNA isolated at 72 h p.i. were aligned to individual viral CDSs or lncRNA sequences and counted. Transcript expression in mutants compared to WT was determined as log_2_-fold change (log_2_FC) values. Differential expression was plotted for all sense transcripts (except RNA2.7) (A), all antisense transcripts (B), regulated sense transcripts only (*q *<* *0.05) (C), and regulated antisense transcripts only (*q *<* *0.05) (D). R and *P* define the strength and significance of the correlation, respectively.

To investigate whether the modulation of mRNA stability applies to viral genes, the relationship between the A+U content of each viral CDS and the magnitude of its transcriptional dysregulation in RNA2.7 mutant-infected cells were assessed. As for cellular mRNAs, A+U-rich viral transcripts were associated with greater downregulation in the RNA2.7 mutants (Fig. 13A). This correlation was moderate for all viral genes (excluding RNA2.7) aggregated (Fig. 13A), and strong for the 22 differentially expressed genes (Fig. 13C). In contrast, the correlation was weak for all antisense transcripts aggregated (Fig. 13B) and negligible for the 29 differentially expressed antisense transcripts (Fig. 13D). Overall, antisense transcripts in WT-infected cells accounted for only 9.2% of all viral transcripts (even excluding RNA2.7) at 72 h p.i.

## DISCUSSION

Given the resources that HCMV devotes to producing RNA2.7, this lncRNA is likely to play important roles during infection. By comparing mutant-infected cells and WT-infected cells, RNA2.7 was shown to be required for broad modulation of gene expression by HCMV, with a large number of mRNAs being regulated late during lytic infection. The 931 transcripts identified were consistently dysregulated in three independent transcriptomic experiments by two different RNA2.7 deletion mutants, the more subtle of which (ΔTATA) differed from WT only by a 6-nt deletion, thus indicating that the phenotype was unlikely to be due to a genetic element other than the RNA2.7 gene. Pathway analysis revealed that 108 of the dysregulated cellular transcripts are associated with cell movement, and 10 of the transcripts in this group were validated in further experiments. Immunoblotting analysis confirmed that protein expression of two CMA genes (TPM3 and STX3) by the RNA2.7 mutants followed the trend observed for the transcripts, and a preliminary proteomics analysis implied this may extend to other CMA genes. The lower proportion of significantly dysregulated CMA genes identified in the proteomics analysis may have been due to the different approaches taken to analyze expression and calculate significance, and perhaps also to the time taken for transcript levels to be represented in protein levels. Live cell tracking of RNA2.7 mutant-infected cells indicated the involvement of RNA2.7 in promoting cell movement late in infection, and morphological analysis experiments further indicated that RNA2.7 is involved in stimulating infected cell detachment. RNA2.7 was also observed to enhance cell-to-cell spread of virus in sparse cell monolayers, probably as a result of its involvement in cell movement. These findings suggest that RNA2.7 may boost intrahost viral dissemination *in vivo*, as cell-to-cell transmission (as opposed to cell-free transfer) is the main mode of HCMV spread (49).

Enhancement of infected cell movement has been observed to play a key role in the spread of other viruses, such as vaccinia virus (50–53). Cell movement is likely to have complex roles, since HCMV infection has been described to increase the motility of monocytes, endothelial cells, the chemokine-mediated migration of smooth muscle cells (54–56) and to modulate the migration of dendritic cells (57), whereas other reports have suggested that HCMV hampers the movement of monocytes and macrophages (58, 59). The underlying mechanisms also appear to be wide-ranging, with modulation of motility being chemokine-dependent or -independent (54, 57–59), mediated through various cellular or viral receptors (54–57), and involving stabilization or dramatic reorganization of the cytoskeleton (56, 58). Immobilization of infected immune cells may be advantageous in circumstances in which it impairs the host antiviral response. Other herpesviruses, such as Kaposi’s sarcoma-associated herpesvirus and Epstein-Barr virus, are also known to promote cell movement, but this has been observed largely in terms of promoting the invasiveness of infected, transformed cells during oncogenesis (60, 61).

A preliminary analysis of growth kinetics showed that the RNA2.7 mutants had a defect when infecting cells that had been glucose-deprived or synchronized by serum starvation. The former property has been attributed previously to a direct interaction of the 5′ one-third of RNA2.7 with GRIM-19 (25). It is possible that the effects of serum starvation have the same cause. However, knockdown experiments indicated that the effects of RNA2.7 on cellular gene regulation are independent of GRIM-19. Analysis of the Δ5′ and Δ3′ mutants showed that the 5′-subdomain, which is sufficient for binding GRIM-19 (27), cannot replace RNA2.7 in the regulation of CMA genes, but, because of low levels of transcription of the residual part of RNA2.7 in the Δ5′ mutant, the role of the 3′-subdomain was not determined. Overall, these results imply that a novel mechanism involving RNA2.7 is responsible for the regulation of cellular transcripts observed in our study. Also, deletion of either subdomain resulted in reduced levels of the residual part of the RNA2.7 transcript, suggesting that sequences within RNA2.7 may stabilize the lncRNA or regulate its transcription. A recent study demonstrated that a CNGGN pentaloop within the 5′-subdomain contributes to RNA2.7 stability (62). This pentaloop interacts with TENT4-ZCCHC14 complex, leading to TENT4-mediated addition of non-A nucleotides to the polyadenylate tail of RNA2.7, thereby conferring protection from deadenylation by the CCR4-NOT (CNOT) complex. However, TENT4 depletion did not alter the level of RNA2.7, suggesting that RNA2.7 utilizes multiple redundant mechanisms to prevent its decay. Self-stabilization is also a characteristic of other herpesvirus lncRNAs: herpes simplex virus 1 LAT (63–67) and Kaposi’s sarcoma-associated herpesvirus PAN (68).

An important caveat of our work is that experiments aimed at determining whether RNA2.7 is able to exert its effects alone and whether the phenotypes of the RNA2.7 mutants could be rescued by expressing RNA2.7 exogenously were compromised by the inability of the expression systems used to match the extremely high levels of RNA2.7 achieved during lytic infection. The foundational study on RNA2.7 function similarly relied on the phenotypes of an RNA2.7 mutant and did not involve rescue or, with one exception, expression of RNA2.7 in isolation (25). In that and subsequent studies (26, 27), RNA2.7 was able to exert its antiapoptotic effect in isolation, whereas our experiments involving inhibition of viral DNA replication indicated that late viral gene products may be involved in the regulation of CMA genes. It is possible that the observed effects of RNA2.7 on ATP production, inhibition of apoptosis and cellular transcription are functionally interdependent (for example, regulation of energy production and differential control of gene expression might form a nexus operating on cell movement), but there are insufficient data to determine whether and to what extent this is the case.

Cellular transcripts upregulated in the presence of RNA2.7, whether associated with cell movement or not, tend to be A+U-rich, which is a characteristic of unstable mRNAs. Moreover, they are enriched in the 3′ UTR in A+U-rich motifs that are targeted by mRNA decay pathways (69). The hypothesis arising from these correlations, that RNA2.7 is required for the upregulation of A+U-rich mRNAs, was supported by the finding that, when mRNA production was inhibited, upregulated transcripts persisted longer in WT-infected cells than RNA2.7 mutant-infected cells. The means by which RNA2.7 achieves transcript stabilization is not known and will be the subject of future studies. Various hypotheses are possible, including that it interacts physically with A+U-rich mRNAs or that it sequesters components of the mRNA decay pathways, perhaps in association with additional viral functions. RNA2.7 is itself A+U-rich and contains five instability-associated motifs (one copy of motif 1 and three copies of motif H1 in the 5′ region, and one copy of motif H1 in the 3′ region), raising the possibility that it interacts directly with mRNA decay pathway components. Moreover, the data indicate that the high level of RNA2.7 may be important for its function. First, although RNA2.7 was readily detectable at 24 h p.i., the transcriptomic analysis did not identify any dysregulated cellular transcripts at that stage in cells infected by the RNA2.7 mutants. Additional RT-qPCR experiments confirmed this for two CMA genes (TPM3 and STX3). Second, ΔRNA2.7 was defective in the promotion of cell rounding, whereas ΔTATA was not, and this partial phenotype may be attributed to the low level of RNA2.7 expressed by the latter. It is also important to register that, although the majority of upregulated transcripts were above average in A+U content, 30.7% were not. It remains to be determined whether these transcripts are also unstable, and whether RNA2.7 might regulate them indirectly via the moderation of A+U-rich transcripts or by another mechanism.

In addition to regulating a large number of cellular mRNAs, RNA2.7 also regulated 22 viral transcripts. The genes encoding these transcripts include both early and late genes and are dispersed throughout the HCMV genome, although 12 occur in clusters of adjacent, similarly oriented genes (UL7, UL8, UL9, and UL10; US7, US8, and US9; UL55, UL56, and UL57; and UL144 and UL145), possibly as a result of 3′-coterminality (the sharing of a 3′ end by a nested set of transcripts) (70, 71). Of the genes in this category with known functions, these appear to be primarily involved in immunomodulation, but this may be due to grouping of such genes in 3′-coterminal families that have arisen by gene duplication. As for cellular transcripts, the susceptibility of viral transcripts to RNA2.7-associated regulation correlated with A+U-richness, suggesting that the effects of RNA2.7 are exerted on all transcripts in the cell. However, modulation of antisense transcription was more modest than that of sense transcription and did not correlate well with A+U-richness. This may have been due to a reduced sensitivity to the relatively low proportion of antisense transcripts or to the perturbation of a complex interdependence between sense and antisense transcription.

Although RNA2.7 generally modulated expression of individual genes with moderate effect, a panoply of cellular and viral genes was targeted. By influencing multiple transcripts involved in the same functional pathway, alone or in concert with other viral functions, RNA2.7 exerted a strong cumulative effect, as demonstrated by the observations on cell motility. Indeed, many HCMV proteins are known to target specific cellular processes in a coordinated fashion, particularly those concerned with manipulating the host immune response (72, 73), and this may be a general strategy employed by HCMV.

## MATERIALS AND METHODS

### Cells and viruses.

Human embryonic kidney cells (293T cells; American Type Culture Collection, ATCC CRL-3216), HFFF2 cells (European Collection of Authenticated Cell Cultures, obtained from Sigma, catalog no. 86031405) and HF-Tet cells (74, 75) were maintained at 37°C in Dulbecco modified Eagle medium supplemented with 10% (vol/vol) fetal bovine serum (FBS), 100 U/ml penicillin, and 100 μg/ml streptomycin and were passaged as monolayers every 3 to 4 days. HFT cells (76) were cultured as monolayers in the same manner with the addition of 50 μg/ml hygromycin B to maintain transduced cells. All cells were tested routinely for the absence of mycoplasma using the MycoAlert mycoplasma detection kit (Lonza).

BACs containing the complete HCMV strain Merlin genome (Merlin-BAC; pAL1111; GenBank accession GU179001.1 [74]) or the Merlin genome with the GFP CDS fused to the 3′-end of the UL36 coding region (UL36-GFP-BAC [34]) were used as parental viruses for constructing RNA2.7 deletion mutants. Two genes (RL13 and UL128) are mutated in Merlin-BAC in order to avoid selecting mutants when virus is reconstituted by transfection into fibroblasts (77). In contrast, viruses reconstituted from UL36-GFP-BAC contain a repaired UL128 and have UL131A under the control of the Tet operator, so that they may be grown stably in repressing HF-Tet cells; the UL128, UL130 and UL131A proteins act as a complex involved in viral entry into nonfibroblast cells, and therefore viruses reconstituted from Merlin-BAC and UL36-GFP-BAC lack this complex. Each deletion mutant was constructed in both Merlin-BAC and UL36-GFP-BAC using BAC recombineering technology (74, 77–79). Briefly, a *kanR/rpsL/lacZ* selectable cassette flanked by sequences directly adjacent to the region to be deleted was created. This cassette was transfected into Escherichia coli SW102 containing Merlin-BAC or UL36-GFP-BAC, allowing it to replace the sequence to be deleted by homologous recombination. Positive clones were selected using kanamycin. A second round of recombineering was then carried out in which the cassette was removed using negative selection with streptomycin, resulting in markerless deletion of the target sequence.

Parental and mutant viruses were reconstituted by transfecting Merlin-BAC-derived BACs into HFFF2 cells or UL36-GFP-BAC-derived BACs into HF-Tet cells using the basic Nucleofector kit for primary mammalian fibroblasts (VPI-1002; Lonza) and electroporation program T-16. Working stocks of virus were prepared and titrated by plaque assay on the appropriate cell line. The complete genome sequence of each virus was determined by Illumina sequencing as described previously (75) and confirmed as being precisely as designed. The sequence data were also shown to contain no mycoplasma sequence contamination.

The parental virus (termed wild-type, WT) and four RNA2.7 deletion mutants (ΔRNA2.7, ΔTATA, Δ5′, and Δ3′) were generated from Merlin-BAC and its derivative BACs. Similarly, the parental virus (termed UL36-GFP-WT) and the four RNA2.7 deletion mutants (UL36-GFP-ΔRNA2.7, UL36-GFP-ΔTATA, UL36-GFP-Δ5′, and UL36-GFP-Δ3′) were generated from UL36-GFP-BAC and its derivative BACs. The coordinates of the deletions in the Merlin genome (GenBank accession AY446894.2) were as follows: ΔRNA2.7 and UL36-GFP-ΔRNA2.7, 2560 to 5050; ΔTATA and UL36-GFP-ΔTATA, 4995 to 5000; Δ5′ and UL36-GFP-Δ5′, 4179 to 4923; and Δ3′ and UL36-GFP-Δ3′, 2560 to 4178.

### Analysis of growth kinetics.

For analysis under standard culture conditions, HFFF2 cells in 6-well plates were infected for 2 h with WT, ΔRNA2.7, or ΔTATA at the specified MOI (quantified in PFU/cell). The medium was then replaced with fresh medium after the cells were washed once with fresh medium. Duplicate 450-μl aliquots of supernatant were harvested every 2 to 3 days and stored at −70°C after pelleting suspended cells and debris by low-speed centrifugation. The pelleted material was added back into the originating well with 1 ml of fresh medium. The stored aliquots of cell-free virus were thawed and titrated on HFFF2 cells. For analysis under conditions of glucose deprivation, HFFF2 cells were incubated in low-glucose (1 g/liter) medium for 48 h prior to infection (MOI of 5), and low-glucose medium was used throughout infection. For analysis under conditions of cell synchronization, HFFF2 cells were synchronized to the G_0_ phase of the cell cycle by incubation in medium lacking serum for 48 h prior to infection (MOI of 5), and the cells were then incubated under standard culture conditions.

### Transcriptomic analysis.

HFFF2 cells in 12-well plates were incubated for 2 h with WT, ΔRNA2.7, or ΔTATA (MOI of 5). The medium was then replaced with fresh medium after washing the cells once with fresh medium. Total cell RNA was harvested at 4, 24, or 72 h p.i. using TRIzol (Invitrogen) and extracted using a Direct-zol kit (Zymo Research) according to the manufacturer’s instructions, including the on-column DNase digestion step. Three independent experiments were performed. Sequencing libraries were prepared from each RNA sample using 500 ng of RNA and a TruSeq stranded mRNA library preparation kit (Illumina, catalog no. 20020594). The libraries were multiplexed and sequenced using a NextSeq500 with 1 × 75-nt high-output kits (Illumina). Each library generated a read data set of about 40 million single-end reads with >91.2% being high quality (>Q30). After demultiplexing, the reads were filtered for quality (>Q20) and trimmed of adapter sequences using Trim Galore v0.4.2.0 (https://github.com/FelixKrueger/TrimGalore).

To analyze the overall viral transcriptome, Bowtie2 v2.3.5 (80) was used to align the reads to the Merlin genome sequence (GenBank accession AY446894.2) lacking the RNA2.7 sequence (nt 2489 to 4969). These results permitted the proportion of viral reads (excluding RNA2.7) to be determined in comparison with the total number of reads. The trimmed reads were also aligned to the complete Merlin genome sequence, and the difference between the number of aligned reads and that in the alignment lacking the RNA2.7 sequence was taken to be the number of RNA2.7 reads. To analyze the transcript levels of individual viral genes, the reads were aligned to the sequences of each of the 170 Merlin CDSs and the four major lncRNA-encoding regions (22) using Bowtie2. The reads in each alignment were sorted into those originating from sense and antisense transcripts, using an in-house script (SamSplit [81]), and realigned with the relevant sequence. The alignments were visualized using Tablet v1.14.11.07 (82). Reads per kilobase per million mapped reads (RPKM) values were determined as described previously (83), thus normalizing the number of mapped reads to CDS length. RNA2.7 reads were again omitted from all transcriptome calculations to facilitate comparison between the WT and RNA2.7 mutant data. Significant changes in viral gene expression (calculated as the log_2_fold change) were determined from the three independent experiments using the R software Limma (*q *<* *0.05) (84).

To analyze the cellular transcriptome, data set quality was confirmed using FastQC (http://www.bioinformatics.babraham.ac.uk/projects/fastqc). The reads were aligned to the human genome sequence (hg19; https://genome.ucsc.edu) using Tophat2 v2.1.1 (85). Genes in RNA2.7 mutant-infected cells that were differentially expressed compared to WT-infected cells were identified using Cufflinks/Cuffdiff v2.2.1 (http://cole-trapnell-lab.github.io/cufflinks/install/) (86). The false discovery rate of differentially expressed transcripts was determined using the Benjamini-Hochberg method, which produced a *q* value for each gene that was considered statistically significant at <0.05. Pathway analysis was performed using Ingenuity Pathway Analysis software (IPA; Qiagen, 2016 release).

### Proteomic analysis.

Confluent HFFF2 cells in 175-cm^2^ flasks were infected for 2 h with WT, ΔRNA2.7, or ΔTATA (MOI of 5). The medium was then replaced with fresh medium after washing the cells once with fresh medium. At 72 h p.i., the cells were washed twice with phosphate-buffered saline (PBS), after which lysis buffer (6 M guanidine and 50 mM HEPES [pH 7]) was added, and the lysed cells were harvested by scraping. The samples were sonicated (30 s on and 30 s off for 10 cycles), and the debris was pelleted by low-speed centrifugation. The cell lysate samples were processed for multistage MS3-based mass spectrometry using isobaric 10-plex tandem-mass tags, and the data were analyzed as described previously (34, 87). A single experiment was conducted, in which 7961 infected cell proteins were identified, including 115 specified by HCMV. Proteins that were differentially expressed in RNA2.7 mutant-infected cells compared to WT-infected cells were identified by calculating Benjamini-Hochberg corrected Significance B values (88), and those with *P < *0.05 were considered to be significant.

### Transcript quantification by PCR.

Confluent HFFF2 or HF-Tet cells in 12-well plates were serum-starved by incubation in the absence of FBS for 48 h prior to infection, in order to synchronize the cell cycle to the G_0_ phase. The cells were then infected for 2 h with WT, ΔRNA2.7, ΔTATA, Δ5′, or Δ3′, or with UL36-GFP-WT, UL36-GFP-ΔRNA2.7, UL36-GFP-ΔTATA, UL36-GFP-Δ5′, or UL36-GFP-Δ3′ (MOI of 5). The medium was then replaced with fresh medium after the cells were washed once with fresh medium. Where appropriate, infected cells were treated with 300 μg/ml PFA (a viral DNA polymerase inhibitor) immediately following the 2-h adsorption period. The infected cells were harvested at 72 h p.i. using TRIzol, and total cellular RNA was isolated using Direct-zol, including the on-column DNase digestion step.

For RT-PCR, reverse transcription was performed using GoScript (Promega) with Oligo-d(T) as the primer, and the resulting cDNA was used as the template for PCRs to amplify specific transcripts along with the GAPDH and G6PD transcripts, which were used to confirm equal loading. Either the Advantage 2 system (Clontech) or the Phusion system (New England Biolabs) was used for PCR (primers are listed in Table S6). For quantitative RT-PCR (RT-qPCR), the one-step RT-qPCR system QuantiTect Virus + ROX Vial kit (Qiagen) was used to quantify the levels of the TPM3, STX3, RNA2.7 and GAPDH transcripts (primers and probes are listed in Table S6), and data were collected using the Applied Biosystems 7500 real-time PCR system (Thermo Fisher). Relative transcript concentrations were determined by comparison to a standard curve and normalized to the level of GAPDH transcript.

### Immunoblotting analysis.

Confluent HFFF2 cells in 12-well plates were serum-starved for 48 h and infected for 2 h with WT, ΔRNA2.7, or ΔTATA (MOI of 5). The medium was then replaced with fresh medium after washing the cells once with fresh medium. At 72 h p.i., cell lysates were harvested using Laemmli sample buffer (89). The proteins were resolved on 4 to 20% (wt/vol) precast TGX polyacrylamide gels (Bio-Rad) using the Mini-PROTEAN Tetra system (Bio-Rad), and transferred to polyvinylidene difluoride membranes (Thermo Scientific). The membranes were blocked with PBS containing 5% (vol/vol) FBS on a roller machine for 1 h, and incubated in PBS containing 5% (vol/vol) FBS, 0.1% (vol/vol) Tween 20 and primary antibodies targeting TPM3 (0.4 μg/ml, HPA009066 [Human Protein Atlas]), STX3 (0.4 μg/ml, HPA002191 [Human Protein Atlas]), GRIM-19 (0.4 μg/ml, HPA041213 [Human Protein Atlas]), or actin (1:4,000 dilution, A1978, clone AC-15; Sigma). The target proteins were visualized by incubating with fluorophore-conjugated secondary antibodies against rabbit (1:10,000, catalog no. 35568; Thermo Fisher) or mouse (1:10,000, SA5-35521; Invitrogen) immunoglobulin in PBS containing 5% (vol/vol) FBS and 0.1% (vol/vol) Tween 20. The signal was detected using an Odyssey CLx instrument (Li-Cor).

### Live cell fluorescence imaging analysis.

Confluent HF-Tet cells in 12-well plates were serum-starved for 48 h prior to infection for 2 h with UL36-GFP-WT, UL36-GFP-ΔRNA2.7, UL36-GFP-ΔTATA, UL36-GFP-Δ5′, or UL36-GFP-Δ3′ (MOI of 5). The medium was then replaced with fresh medium after washing the cells once with fresh medium. Where appropriate, infected cells were treated with 300 μg/ml PFA immediately after the 2-h viral adsorption period. The medium was then replaced with fresh medium immediately before carrying out automated time-lapse, multiposition, dual-channel microscopy at 48 to 120 h p.i. at ×20 magnification using the Cell Observer live cell imaging system (Zeiss) fitted with the CO_2_ module, Temp module S and Incubator XLmulti S1. Images of five random fields of view per well with duplicate wells per condition were obtained every 10 min using both the brightfield and GFP channels. GFP expression was confirmed in all cells and tracked using the Fiji plug-in TrackMate v3.4.2 (90). GFP-expressing cells were identified by the LoG detector; the estimated cell diameter was set at 0.002 cm. Only spots of quality >0.3 were accepted. The Linear Assignment Problem (LAP) algorithm was used to create the cell movement tracks, for which the maximum frame-to-frame linking distance was 0.002 cm, the maximum track distance for gap-closing was 0.003 cm, the maximum gap was 2 frames, and the maximum distance permitted for track segment splitting was 0.002 cm. Only tracks with at least 15 successful detections of the same cells and a quality standard deviation of >0.1 were included in the analysis. An average of 756 tracks per condition were examined in each experiment.

### Analysis of infected cells in individual plaques.

Confluent HF-Tet cells in 12-well plates were serum-starved for 48 h prior to infection for 2 h with UL36-GFP-WT, UL36-GFP-ΔRNA2.7, or UL36-GFP-ΔTATA (MOI of 5). The medium was then replaced with fresh medium after washing the cells once with fresh medium. The infected cells were trypsinized at 72 to 96 h p.i. and resuspended in medium. Dispersal into isolated cells was confirmed microscopically, and cell density was determined twice using a hemocytometer. About 60 cells were used to seed plaques in partially (25%) or fully confluent HF-Tet cells in 6-well plates in the presence of 50 μg/ml Cytotect anti-HCMV antibody (Biotest) (91). The number of infected (GFP-expressing) cells in each of the plaques in duplicate wells was counted after 48 h had elapsed, resulting in the assessment of 70 to 100 plaques per virus in each experiment.

### Generation of GRIM-19 knockdown cell lines.

Derivatives of the TRC lentiviral cloning vector (92) encoding shRNAs against three different regions of the GRIM-19 gene (TRCN000006492, TRCN0000064929, and TRCN0000236379; Sigma) or a nontargeting control shRNA (SHC002; Sigma) were transfected using Lipofectamine 3000 (Sigma) into 293T cells with the Δ8.9 and pVSV-g plasmids to produce infectious lentiviruses (93). The lentiviruses were used to transduce HFT cells, and successfully transduced cells were selected and maintained by incubation with medium containing 1 or 0.5 μg/ml puromycin.

### Analysis of RNA stability motifs and nucleotide composition.

Enrichment of RNA stability motifs in query data sets of human mRNAs was tested using a randomization approach. The query data set was either the 146 downregulated genes, the 785 upregulated genes, or the subset of 99 upregulated CMA genes. Human genome annotation hg19 was used. No genes were excluded from the CMA gene query data set, but five downregulated transcripts and eight upregulated transcripts (see Table S5B) were excluded from the total query data set because the genes lacked protein identities in the human genome database. Nonetheless, nearly all regulated cellular genes (98.6%) were included in the analysis.

The total number of motifs in all sequences was counted in each query data set using a sliding window of a size equal to that of the motif, shifted in 1-nt increments. For genes with multiple transcript variants, the longest variant was used. The mean number of each motif per sequence in a query data set was calculated for the entire mRNA and also for separate regions: the 5′ UTR, the 3′ UTR, and the CDS. This analysis was repeated with 1,000 data sets containing a number of sequences equal to that in the query data set, each randomly sampled from the human genome database (GRCh38.p12 [https://www.ensembl.org/info/data/ftp/index.html]). The mean number of each motif per sequence in each randomly sampled data set was compared with that from the query data set. The difference was considered to be significant if this number was higher or lower in >950/1000 comparisons (i.e., *P < *0.05). All A+U analyses were performed in the same manner by determining the mean values for the longest transcript variant.

### Data availability.

Transcriptomic sequence read data sets were deposited in the NCBI Sequence Read Archive under the following accession numbers: WT 4 h, SRX8009962, SRX8009951, and SRX8009950; ΔRNA2.7 4 h, SRR12424745, SRR12424744, and SRR12424753; ΔTATA 4 h, SRR12424752, SRR12424751, and SRR12424750; WT 24 h, SRX8009953, SRX8009952, and SRX8009976; ΔRNA2.7 24 h, SRR12424747, SRR12424749, and SRR12424748; ΔTATA 24 h, SRR12424746, SRR12424743, and SRR12424742; WT 72 h, SRX8009963, SRX8009961, and SRX8009960; ΔRNA2.7 72 h, SRR12424759, SRR12424757, and SRR12424758; and ΔTATA 72 h, SRR12424756, SRR12424755, and SRR12424754. Proteomic data sets were deposited in the ProteomeXchange with identifier PXD022277. The Python script for analyzing stability motif enrichment is available in Github (https://github.com/salvocamiolo/stabilityMotifs.git).
